# Efficient CO_2_ capture by tertiary amine-functionalized ionic liquids through Li^+^-stabilized zwitterionic adduct formation

**DOI:** 10.3762/bjoc.10.204

**Published:** 2014-08-21

**Authors:** Zhen-Zhen Yang, Liang-Nian He

**Affiliations:** 1 State Key Laboratory and Institute of Elemento-Organic Chemistry, Collaborative Innovation Center of Chemical Science and Engineering (Tianjin), Nankai University, Tianjin 300071, P. R. China

**Keywords:** carbon capture and sequestration, CO_2_ chemistry, coordination effect, ionic liquid, polyethylene glycol, zwitterionic adducts

## Abstract

Highly efficient CO_2_ absorption was realized through formation of zwitterionic adducts, combining synthetic strategies to ionic liquids (ILs) and coordination. The essence of our strategy is to make use of multidentate cation coordination between Li^+^ and an organic base. Also PEG-functionalized organic bases were employed to enhance the CO_2_-philicity. The ILs were reacted with CO_2_ to form the zwitterionic adduct. Coordination effects between various lithium salts and neutral ligands, as well as the CO_2_ capacity of the chelated ILs obtained were investigated. For example, the CO_2_ capacity of PEG_150_MeBu_2_N increased steadily from 0.10 to 0.66 (mol CO_2_ absorbed per mol of base) through the formation of zwitterionic adducts being stabilized by Li^+^.

## Introduction

Carbon capture and sequestration (CCS) from flue gas formed by combustion of fossil fuel is a critical part of efforts directed towards the stabilization of atmospheric greenhouse gas levels [1]. In recent years, there has been intense research worldwide aimed at the development of various processes and technologies for efficient CO_2_ capture. These efforts include the development of liquid and solid absorbents and membranes [2–7].

**Scheme 1:**
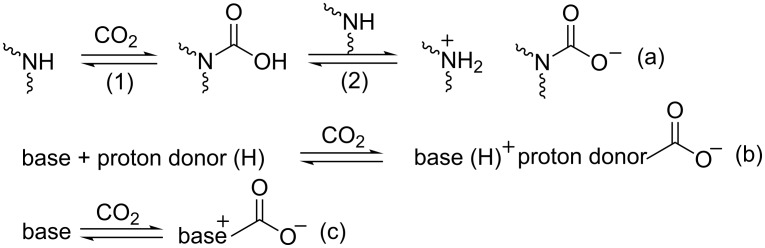
Reactions of CO_2_ with amino-group containing absorbents (a), base/proton donor binary system (b) or strong organic base (c).

Ionic liquids (ILs), which have attractive properties such as negligible vapor pressure, a wide liquid temperature ranges, good thermal stability, high ionic conductivity, and versatile solvation properties [8–11], can be designed for task-specific applications through the smart choice of the respective cations and/or anions. Application fields include green solvents for synthesis [9,12–15], efficient catalysts in organic synthesis [2,16–17], media for advanced separation [18–19], novel electrolytes for energy applications [20–21], and efficient absorbents for gas separation [2,22–24]. In particular, amino-functionalized IL [APBIm][BF_4_] (1-aminopropyl-3-butylimidazolium tetrafluoroborate) and ILs being composed of amino acid (AA) anions and phosphonium or ammonium cations were developed for efficient CO_2_ chemisorption [23,25–30]. Binary absorbents derived from superbases together with various non-volatile weak proton donors such as hydroxy-functionalized ILs, imidazolium ILs, fluorinated alcohol, imidazole and phenol, were also found to be efficient liquid absorbents allowing for reversible CO_2_ chemisorption [31–35]. In general, two absorbent molecules are involved to react with one CO_2_ molecule generating ammonium carbamate (Scheme 1a) or ammonium alkyl formate (Scheme 1b). Hence, increasing the 1:2 (CO_2_:absorbent molecule) stoichiometry for the CO_2_ capacity to 1:1 is an essential prerequisite for a breakthrough in absorption techniques [23]. In this respect, task-specifically designed absorbents have been successfully synthesized from AAs and applied for 1:1 CO_2_ capture through a carbamic acid formation pathway (Scheme 1a, step 1). Notably, equimolar CO_2_ absorption was obtained using task-specific ionic liquids (TSILs) with the phosphonium cation containing long alkyl chains and anions derived from AAs (prolinate and methioninate) [36], or AA salts with bulky N-substituents in polyethylene glycol (PEG) solution [37]. However, procedures for the preparation of ILs usually include complicated purification procedures or the use of volatile organic solvents (e.g., toluene, acetonitrile). Recently, Wang et al. developed novel alkanolamine-based ILs through multi-dentate cation coordination between alkanolamine and Li^+^ for reversible CO_2_ capture, by simple mixing of equimolar amounts of alkanolamines with LiNTf_2_ [38]. The strong complexation of alkali metal cations by crown ethers could be used to achieve equimolar CO_2_ absorption in systems containing crown ethers and easily available alkali metal salts of amino acids, resulting in the respective carbamates (onium salts) [39].

As previously reported, strong amidine and guanidine bases such as 1,5,7-triazabicyclo[4.4.0]dec-5-ene (TBD) can form the base-CO_2_ zwitterionic adduct in a 1:1 manner under strictly anhydrous conditions (Scheme 1c) [40–41]. Herein, we present such a method combining the formation of ILs and coordination to achieve equimolar CO_2_ capture through zwitterionic adduct formation. The essence of our strategy is to make use of the multisite coordination interaction between Li^+^ and organic bases or PEG-functionalized organic bases. The readily prepared ILs were reacted then with CO_2_ to form intramolecular zwitterionic adducts.

## Results and Discussion

Taking PEG_150_MeTMG as a neutral ligand (Table 1), coordination effects of various lithium salts were investigated (Table S1, Supporting Information File 1). Only LiSO_3_CF_3_ and LiNTf_2_ formed complexes with PEG_150_MeTMG through multisite coordination. Subsequently, different neutral ligands with alkyl chains or PEG chain were selected to evaluate the effect of chelating with LiNTf_2_ on the physicochemical properties of the resulting ILs as well as the CO_2_ capacities (Table 1).

**Table 1:** Stability (Δ*H*_f_) of the cations derived from coordination of Li^+^ in LiNTf_2_ with various neutral ligands and CO_2_ capacity of the derived ionic liquids^a^.

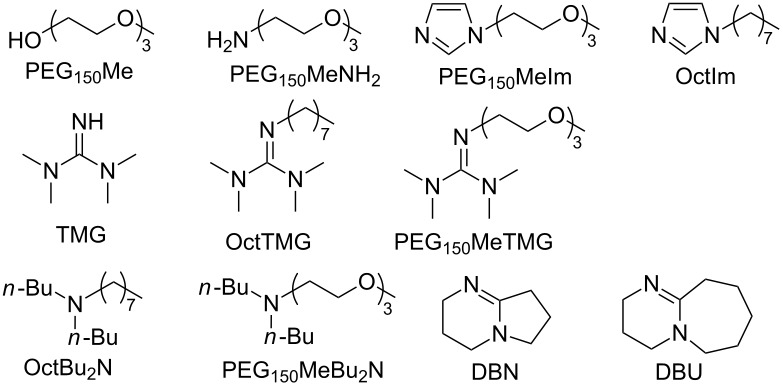			

Entry	Ionic liquid	Δ*H*_f_/kcal mol^−1 b^	CO_2_ capacity^c^

1	[PEG_150_MeLi][NTf_2_]	−100.94	0.09 (0.9%)
2	[OctImLi][NTf_2_]	−56.49	0.11 (1.0%)
3	[PEG_150_MeImLi][NTf_2_]	−91.17	0.16 (1.4%)
4	[PEG_150_MeNH_2_Li][NTf_2_]	−89.39	0.45 (4.4%)
5	[TMGLi][NTf_2_]	−41.59	0.65 (7.1%)
6	[OctTMGLi][NTf_2_]	−47.79	0.80 (6.8%)
7	[PEG_150_MeTMGLi][NTf_2_]	−106.56	0.89 (7.1%)
8	OctBu_2_N/LiNTf_2_	–	–
9	PEG_150_MeBu_2_N	–	0.10 (1.6%)
10	[PEG_150_MeBu_2_NLi][NTf_2_]	−96.26	0.66 (5.2%)
11	[PEG_150_MeBu_2_NLi][SO_3_CF_3_]	−96.26	0.61 (6.2%)
12	[DBULi][NTf_2_]	−60.22	0.50 (5.0%)
13	[DBNLi][NTf_2_]	−60.50	0.75 (8.0%)

^a^Ionic liquids were prepared by mixing of a neutral ligand with LiNTf_2_ in 1:1 molar ratio. CO_2_ absorption was carried out at 25 °C and absorption equilibrium was reached within 20 min. ^b^Energy of the gas phase reaction between the neutral ligand and Li^+^, was calculated with DFT, using the B3PW91 functional with the 6-311++G (d,p) basis set as implemented in the Gaussian 09 program package. ^c^Mol of CO_2_ captured per mol of ionic liquid. Results in bracket were grams of CO_2_ absorbed per gram of absorbent.

**Figure 1:**
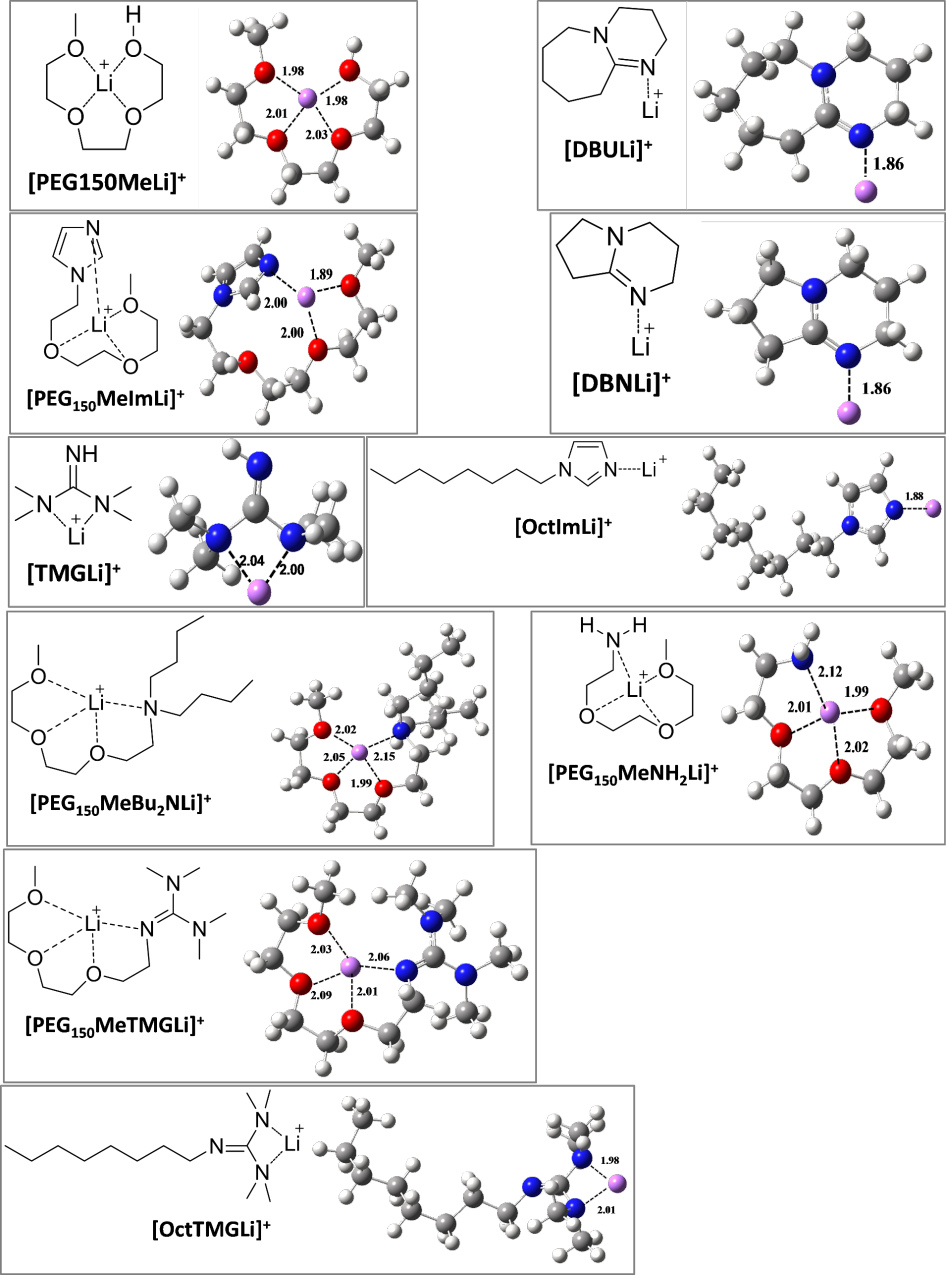
Typical optimized structures of complex cations derived from chelation between Li^+^ and neutral ligands. H: white, C: grey, O: red, N: blue, Li: purple. Bond lengths are in Å.

Typical optimized structures of cations derived from chelation between neutral ligands and Li^+^ are shown in Figure 1. The geometry optimizations were carried out by performing DFT calculations. Four O atoms in PEG_150_Me chelate with Li^+^ in a quasi-crown ether manner. For neutral ligands with PEG chain (PEG_150_MeIm, PEG_150_MeNH_2_, PEG_150_MeTMG and PEG_150_MeBu_2_N), all the O and N atoms coordinate with Li^+^ in a quasi-aza-crown ether fashion. OctIm, OctTMG, TMG, DBU and DBN generate complexes whereby Li^+^ is bound only to N atoms. In contrast, the coordination ability of the N atom in OctBu_2_N is not strong enough to form a homogeneous chelated IL with LiNTf_2_.

Calculation of the energy of the gas phase reaction between neutral ligands and Li^+^ gave a value for the enthalpy change in the range of −41.59 to −106.56 kcal mol^−1^ (Table 1, entries 1–11), indicating that the formation of chelated ILs is feasible.

In addition, PEG-functionalization of the organic base enhanced the complexation ability. For example, Δ*H*_f_ decreased from −56.49 kal mol^−1^ (OctIm) to −91.17 kcal mol^−1^ (PEG_150_MeIm) for imidazole (Table 1, entry 2 vs 3), from −47.79 kcal mol^−1^ (OctTMG) to −106.56 kcal mol^−1^ (PEG_150_MeTMG) for guanidine (Table 1, entry 6 vs 7), and from no complexation (OctBu_2_N) to −96.26 kal mol^−1^ (PEG_150_MeBu_2_N) for tertiary amine (Table 1, entry 8 vs 9).

**Figure 2:**
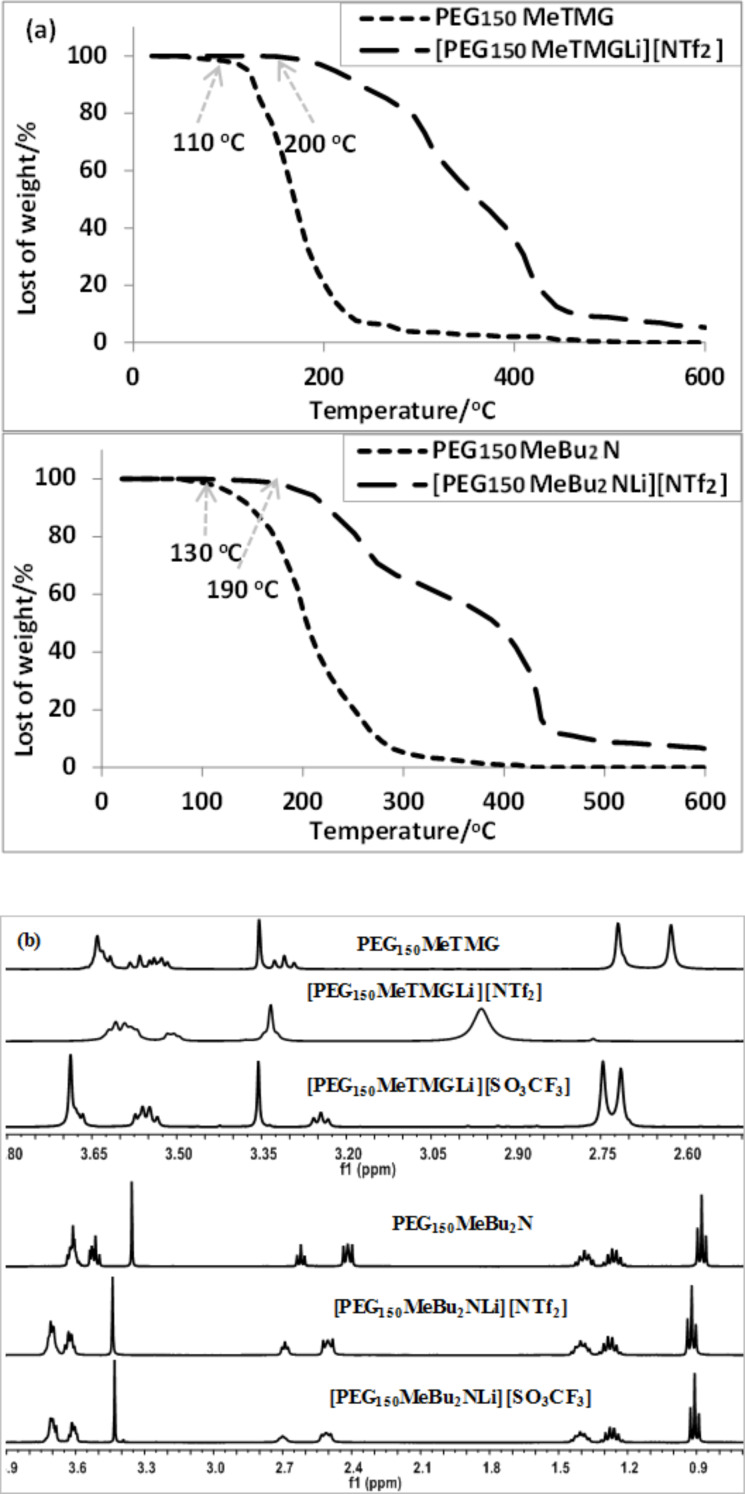
(a) Comparison of the thermal stability between the neutral ligands and the corresponding chelated ionic liquids after coordinating with LiNTf_2_, being detected by TGA; (b) ^1^H NMR (CDCl_3_, 400 MHz) spectrum of PEG_150_MeTMG and PEG_150_MeBu_2_N before and after reacting with lithium salts (LiNTf_2_ and LiSO_3_CF_3_).

The structures of the chelated ILs were confirmed by thermogravimetric analysis (TGA), NMR (see Supporting Information File 1, Figure S2), in situ FTIR under CO_2_ pressure and mass spectrometry. The thermal stability of the chelated ILs was strongly increased compared to the corresponding neutral ligands. The decomposition temperature increased by 90 °C and 60 °C for PEG_150_MeTMG and PEG_150_MeBu_2_N, respectively, after coordination with LiNTf_2_ (Figure 2a).

**Figure 3:**
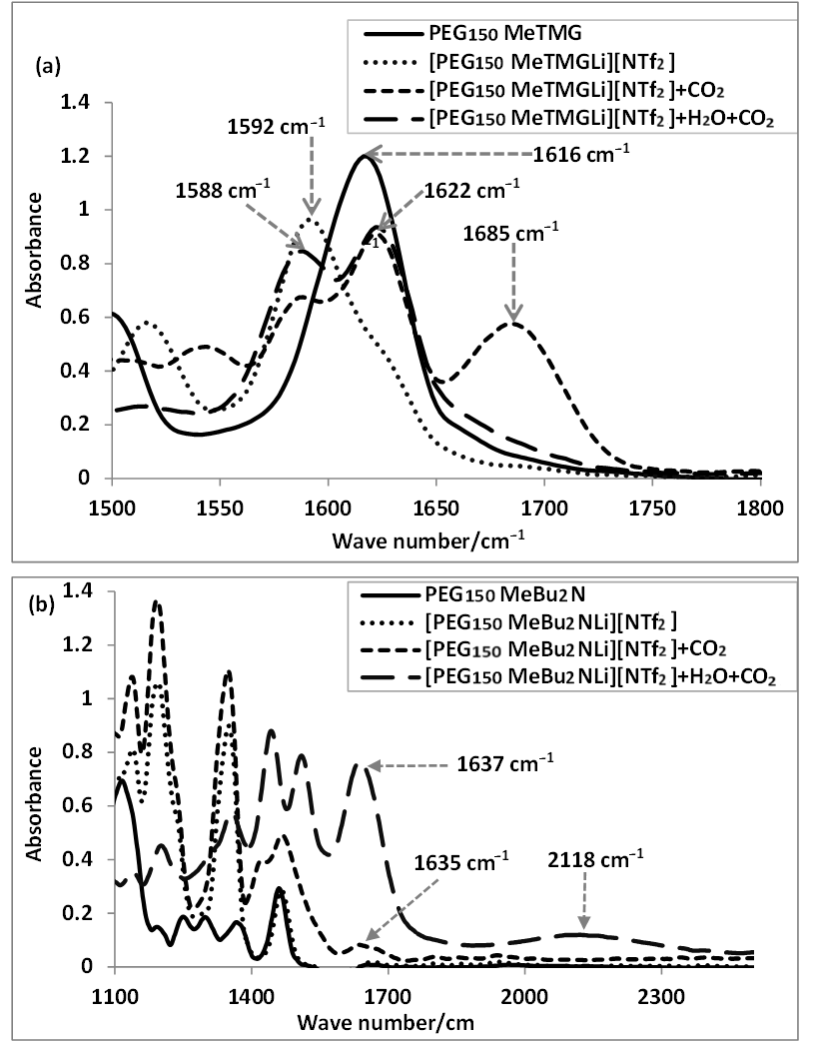
In situ FTIR spectra of neutral ligands and the corresponding chelated ionic liquids after reaction with LiNTf_2_, as well as the reaction mixture after CO_2_ absorption in the absence or presence of water.

In the ^1^H NMR spectrum, the proton signals of the four methyl groups in guanidine of PEG_150_MeTMG shifted from 2.63–2.72 ppm to 2.96 ppm after reacting with equimolar amounts of LiNTf_2_ (Figure 2b). The corresponding anion CF_3_SO_3_^−^, the twelve protons moving only from 2.63–2.72 ppm to 2.71–2.75 ppm, indicate that there is only little influence of the anion on the nature of the coordinative bond. In the case of tertiary amines (e.g., PEG_150_MeBu_2_N), all the protons shifted downfield after forming the chelated IL with LiNTf_2_. Changing the anion to CF_3_SO_3_^−^ had negligible influence on the coordination ability. All the neutral ligands shown in Table 1 gave a downfield shift in the ^1^H NMR spectrum after chelating with Li^+^, in accordance with the electron density decreasing through coordination. Furthermore, the formation of chelated ILs was also verified by ESI-MS (*m*/*z* = 268.27 for [PEG_150_MeTMGLi]^+^ and *m*/*z* = 282.32 for [PEG_150_MeBu_2_NLi]^+^) (Figure S3, see Supporting Information File 1). In the in situ FTIR spectrum, the C=N absorption band of the neutral ligand PEG_150_MeTMG was shifted from 1616 cm^−1^ to 1592 cm^−1^ after reacting with equimolar amounts of LiNTf_2_ as depicted in Figure 3a.

**Figure 4:**
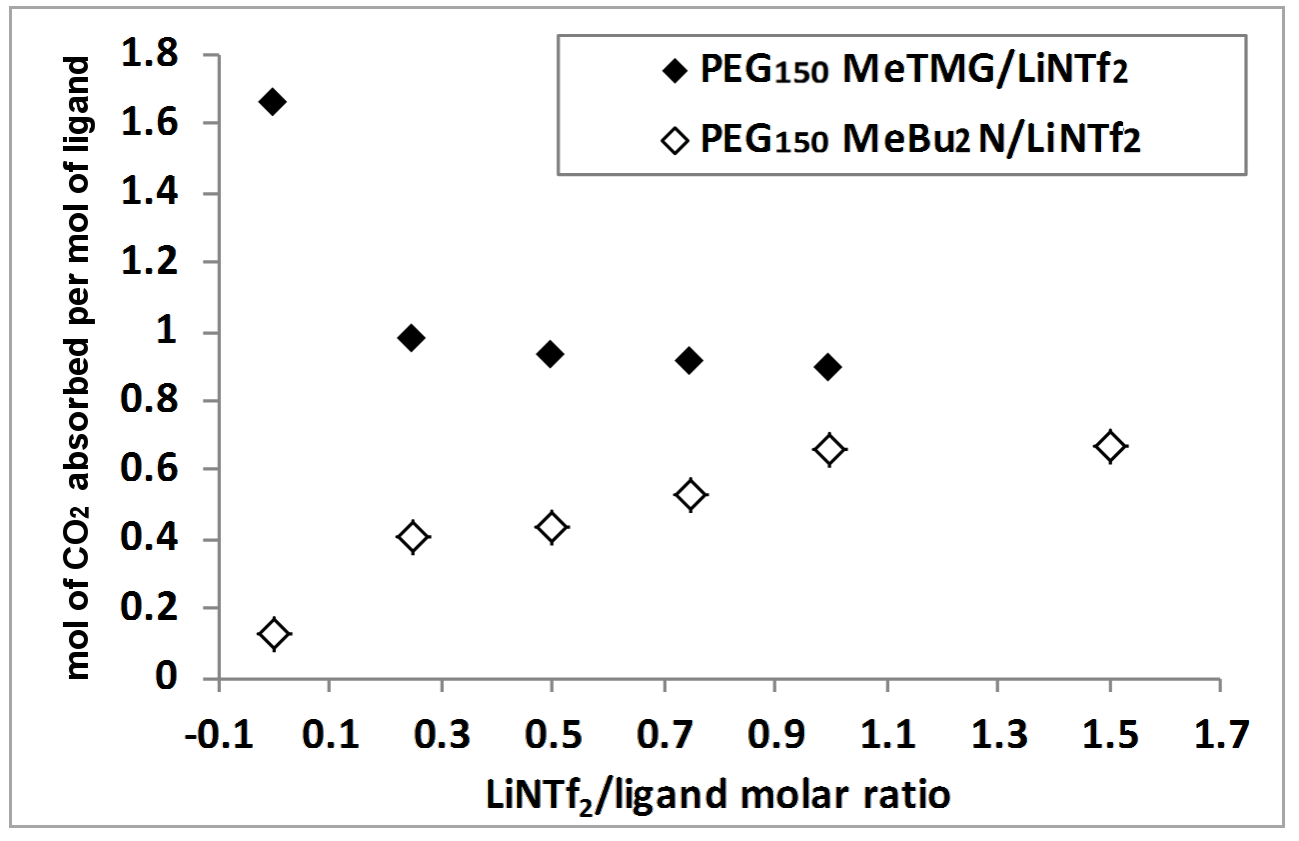
Influence of the ratio of LiNTf_2_/neutral ligands (PEG_150_MeTMG and PEG_150_MeBu_2_N) on the CO_2_ capacity of the coordinating mixtures.

The effect of various chelated ILs on CO_2_ absorption was subsequently examined using LiNTf_2_ as coordinating reagent. The CO_2_ absorption capacity, defined as mol of CO_2_ captured per mol of IL, was estimated from the weight increase of the reaction mixture. As shown in Table 1, ILs of weak basicity, such as [PEG_150_MeLi][NTf_2_], [OctImLi][NTf_2_] and [PEG_150_MeImLi][NTf_2_] showed a poor CO_2_ sorption capacity, implying that only physical interactions with CO_2_ were present (Table 1, entries 1–3). The primary amine-functionalized IL [PEG_150_MeNH_2_Li][NTf_2_] gave rise to CO_2_ uptake approaching 1:2 stoichiometry (Table 1, entry 4) as expected from the proposed mechanism for the formation of ammonium carbamate as shown in Scheme 1a [38]. Notably, the CO_2_ capacity of guanidine-functionalized ILs increased in the order of [TMGLi][NTf_2_] (0.65) < [OctTMGLi][NTf_2_] (0.80) < [PEG_150_MeTMGLi][NTf_2_] (0.89 mol CO_2_ absorbed per mol of base) (Table 1, entries 5–7), indicating that the CO_2_-philic nature of the PEG chain facilitates CO_2_ sorption. Generally, anhydrous tertiary amines absorb CO_2_ only under high CO_2_ pressures to form instable zwitterionic alkylcarbonate salts (Table 1, entry 9) [42]. [PEG_150_MeBu_2_NLi][NTf_2_] and [PEG_150_MeBu_2_NLi][SO_3_CF_3_] were able to rapidly reach 0.66 and 0.61 CO_2_ capacity, respectively. Thus, tertiary amino-functionalized ILs with multidentate cation coordination have a much better performance probably due to the formation of a zwitterionic adduct being stabilized by Li^+^ (Table 1, entries 10 and 11). Indeed, the CO_2_ absorption capacity of [PEG_150_MeBu_2_NLi][NTf_2_] increased steadily from 0.10 to 0.66 mol CO_2_ absorbed per mol of base when the molar ratio of LiNTf_2_/PEG_150_MeBu_2_N was varied from 0 to 1. When the molar ratio was increased to 1.5, no further promotion of the CO_2_ capacity was observed (Figure 4). In contrast, when PEG_150_MeTMG was employed as the neutral ligand, the CO_2_ capacity decreased from 1.66 to 0.89 mol CO_2_ absorbed per mol of base as the molar ratio of LiNTf_2_/PEG_150_MeTMG was increased from 0 to 1, probably due to decreased basicity of guanidine after coordinating with Li^+^. At last, ILs [DBULi][NTf_2_] (0.50) and [DBNLi][NTf2] (0.75 mol CO_2_ absorbed per mol of base) had a CO_2_ capacity below 1:1 stoichiometry expected from the proposed mechanism (Scheme 1c), owing to highly increased viscosity after CO_2_ absorption (Table 1, entries 12 and 13). Compared on a weight basis, a CO_2_ capacity of 5.0 wt % to 8.0 wt % was obtained with ILs from neutral ligands/LiNTf_2_ (Table 1, entries 5–7 and 10–13). This is much higher than for the conventional IL 1-hexyl-3-methylimidazolium hexafluorophosphate (0.0881 wt %) [43], and comparable to amino-functionalized imidazolium-based IL (7.4 wt %) [23] and ILs derived from amino acids [24]. Hence, our IL system has the potential to be utilized for industrialized CO_2_ absorption processes.

The in situ FTIR spectrum of [PEG_150_MeTMGLi][NTf_2_] and [PEG_150_MeBu_2_NLi][NTf_2_] before and after reaction with CO_2_ are shown in Figure 3. For [PEG_150_MeTMGLi][NTf_2_] as absorbent, the C=N stretching band at 1592 cm^−1^ shifted to 1622 cm^−1^ after reaction with CO_2_. A characteristic peak centered at 1685 cm^−1^ is assigned to the stretching vibration of the carbonyl group in the zwitterionic alkyl carbamate, which is quite different from that in HCO_3_^−^ (1588 cm^−1^). For the reaction of [PEG_150_MeBu_2_NLi][NTf_2_] with CO_2_, a new band at 1635 cm^−1^ was assigned to the stretching vibration of the C=O bond in the zwitterionic product. In addition, a distinct broad band at around 2118 cm^−1^ corresponding to the ammonium cation was observed in the presence of water.

**Figure 5:**
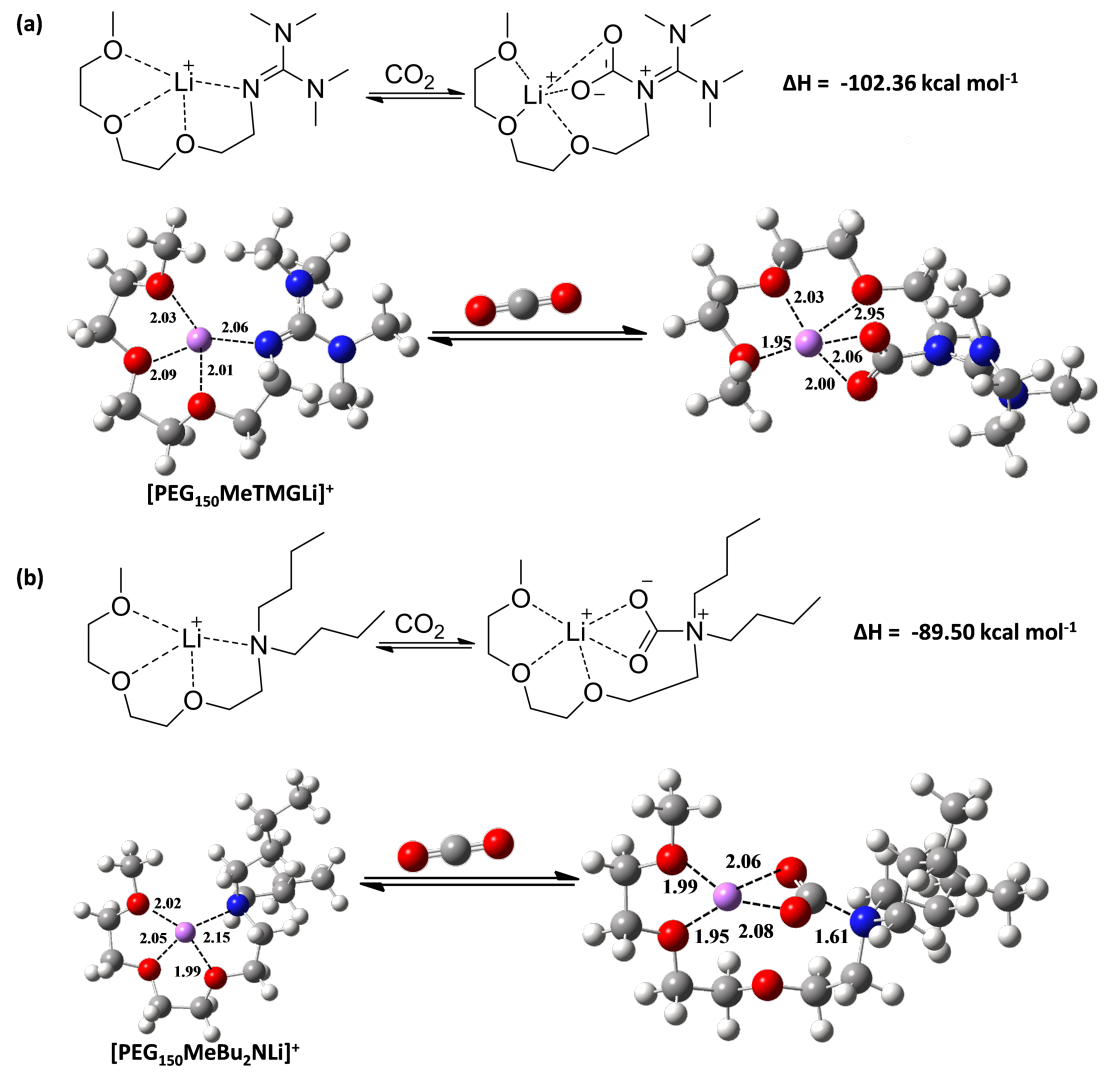
The quantum chemistry calculations (enthalpy changes) of the reaction between CO_2_ and [PEG_150_MeTMGLi]^+^ (a) or [PEG_150_MeBu_2_NLi]^+^ (b); H: white, C: grey, O: red, N: blue, Li: purple. Bond lengths are in Å.

To gain deeper insight into the reaction mechanism of CO_2_ absorption with chelated ILs, DFT calculations were carried out. We performed geometry and energy optimizations for the free [BaseLi]^+^ cation, free CO_2_ and the [BaseLi]^+^ + CO_2_ complex. As shown in Figure 5, the chelated ILs react with CO_2_ through nucleophilic attack by the N atom, to form the zwitterionic adducts, which are stabilized through coordination with Li^+^. In addition, formation of the zwitterionic alkyl carbamate is calculated to be associated with an enthalpy changes of −102.36 kcal mol^−1^ and −89.50 kcal mol^−1^ for [PEG_150_MeTMGLi]^+^/CO_2_ and [PEG_150_MeBu_2_NLi]^+^/CO_2_, respectively, indicating that the absorption process is thermodynamically favourable.

## Conclusion

In summary, efficient CO_2_ capture was achieved through formation of zwitterionic adducts with readily synthesized chelated ILs. Multisite coordination interaction between the Li^+^ cation and organic bases or PEG-functionalized organic bases is thought to be responsible for forming the amidine, guanidine or tertiary amine-functionalized ILs, after reaction with CO_2_ to form zwitterionic adducts in a 1:1 manner. Coordination effects between various lithium salts and neutral ligands, and the CO_2_ capacity of the obtained chelated ILs were investigated. Indeed, the thermal stability and the CO_2_ capacity of the neutral ligands (e.g., PEG_150_MeBu_2_N) was highly increased after coordination with lithium salts to form chelated ILs (e.g., [PEG_150_MeBu_2_NLi][NTf_2_]).

## Experimental

### Materials

All reagents used in this work were purchased from Sigma-Aldrich and used without further purification. CO_2_ with a purity of 99.999% was obtained commercially.

### Experimental methods

^1^H NMR spectra were recorded on a Brucker 400 spectrometer in CDCl_3_. Residual CHCl_3_ (7.26 ppm) was used as internal reference. ^13^C NMR spectra were recorded at 100.6 MHz in CDCl_3_. Residual CHCl_3_ (77.0 ppm) was used as internal reference. In situ FTIR spectra were collected on a Mettler Toledo React IR ic10, which was equipped with a diamond ATR probe, using an ic IR analysis system. The probe was placed into the absorption mixture. Spectra were collected in situ during CO_2_ absorption, while the mixture was stirred continuously using a magnetic stir bar. ESI-MS spectra were recorded on a Thermo Finnigan LCQ Advantage spectrometer in ESI mode at a spray voltage of 4.8 kV.

### General procedure for CO_2_ absorption

The CO_2_ absorption procedure was analogous to the CO_2_/SO_2_ absorption procedure we had reported before [35,44]. In a typical procedure, the CO_2_ capture was carried out in a 10 mL Schlenk flask. The absorbents were charged into the reactor at room temperature. Then, the air in the flask was replaced by passing CO_2_ through a needle, which was inserted into the bottom of the flask. The absorption was conducted at 25 °C with a CO_2_ flow rate of 0.1 L/min. The amount of CO_2_ absorbed was determined by following the weight of the mixture with an Analytical Balance. Data points were taken with an accuracy of ±0.0001 g every five minutes. Absorption/desorption was determined for at least three cycles.

## Supporting Information

File 1 General experimental methods, synthesis and characterization of the neutral ligands, lithium salts and the corresponding chelated ionic liquids.
