# Supplementary material for: Efficient CO2 capture by tertiary amine-functionalized ionic liquids through Li+-stabilized zwitterionic adduct formation
Source: Beilstein J Org Chem. 2014 Aug 21;10:1959–66. doi: 10.3762/bjoc.10.204 (PMC4168910; doi:10.3762/bjoc.10.204)
Supplement: File 1 — General experimental methods, synthesis and characterization of the neutral ligands, lithium salts and the corresponding chelated ionic liquids. [file Beilstein_J_Org_Chem-10-1959-s001.pdf]

Supporting Information

for

Efficient CO_2 capture by tertiary amine-functionalized ionic liquids through Li^+-stabilized zwitterionic adduct formation

Zhen-Zhen Yang, Liang-Nian He*

Address: State Key Laboratory and Institute of Elemento-Organic Chemistry, Collaborative Innovation Center of Chemical Science and Engineering (Tianjin), Nankai University, Tianjin 300071, P. R. China.

Email: Liang-Nian He* - heln@nankai.edu.cn

*Corresponding author

General experimental methods, synthesis and characterization of the neutral ligands, lithium salts and the corresponding chelated ionic liquids

Table of contents

page

1. Synthesis and characterization of the neutral ligands and lithium salts **S2**

2. Coordination effect of different lithium salts with $PEG_{150}MeTMG$ (Table S1) **S3**

3. ^1H NMR ($CDCl_3$, 400 MHz) spectrum of the neutral ligands and the corresponding chelate ionic liquids (Figure S1) **S4**

4. ESI spectrum of $PEG_{150}MeTMG$, $PEG_{150}MeBu_2N$ before and after coordinating with $LiNTf_2$ (Figure S2) **S7**

5. References **S9**

6. NMR characterization and charts of all the neutral ligands and chelate ionic liquids **S9**

1. Synthesis and characterization of the neutral ligands and lithium salts

Synthesis of OctIm, PEG150MeIm and PEG150MeCl were performed in the same manner as described in the literature.[1]

Synthesis of PEG_{150}MeTMG, OctTMG, $PEG_{150}MeBu_2N$ and $OctBu_2N$ was in an analogous manner as reported in the literature.[2]

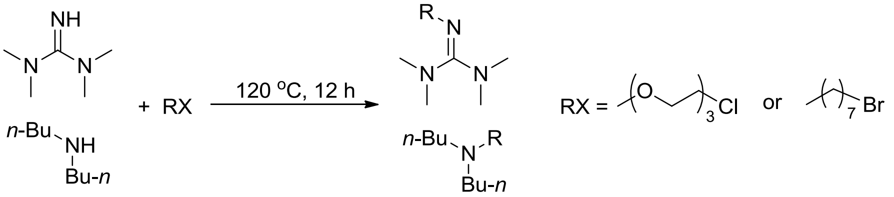

In a dry flask, 1,1,3,3-tetramethylguanidine (TMG) or n-Bu_2NH (30 mmol) and xylene (1 mL) were mixed together. After being evacuated and purged with N_2 five times, the system was heated to 120 °C and maintained at this temperature for 2 h. Then PEG_{150}MeCl or 1-bromooctane (15 mmol) was added dropwise. After being stirred at 120 °C for 12 h under N_2 atmosphere, the resulting mixture was allowed to cool to room temperature. The formed salt precipitates were removed by filtration. The filtrate was distilled under reduced pressure and the product (PEG_{150}MeTMG, OctTMG, $PEG_{150}MeBu_2N$ and $OctBu_2N$) obtained as colorless liquid.

Synthesis of $LiOCH_2CF_3$ and $LiOCH(CF_3)_2$

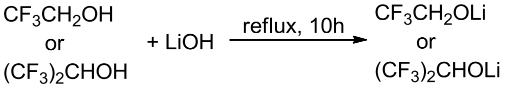

LiOH (10 mmol) and $HOCH_2CF_3$ or $HOCH(CF_3)_2$ (30 mmol) were stirred at reflux for 10 h, and then excess fluoro-substituted alcohol was removed on a rotator evaporator under reduced pressure. The residue was dried in vacuum to give $LiOCH_2CF_3$ or $LiOCH(CF_3)_2$ as a white solid.

$LiOCH(CF_3)_2$

^{1}H NMR (D_2O, 400 MHz) δ 4.35-4.42 (m, 1H); ^{19}F NMR (D_2O, 377 MHz) δ -76.13; ^{13}C NMR (D_2O, 100.6 MHz) 128.4, 125.6, 122.8, 119.9, 73.4, 73.1, 72.8, 72.5, 72.2.

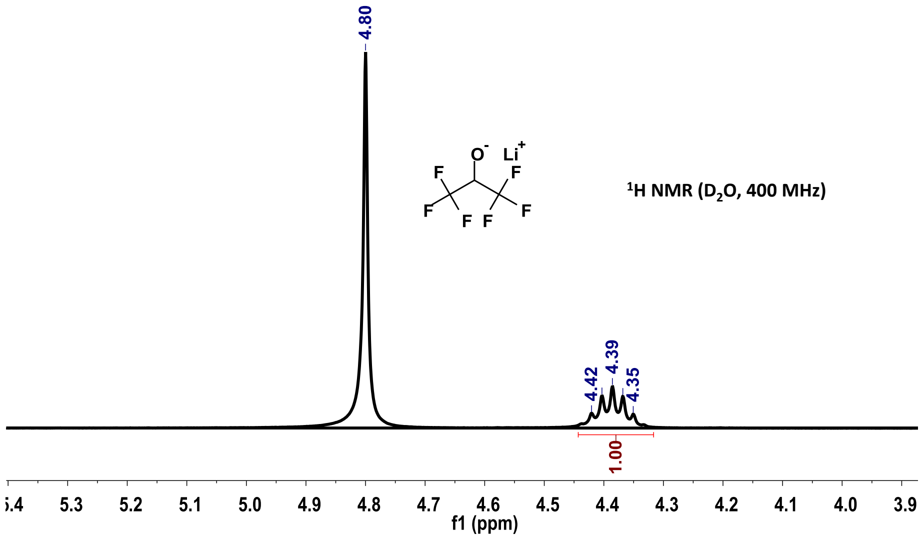

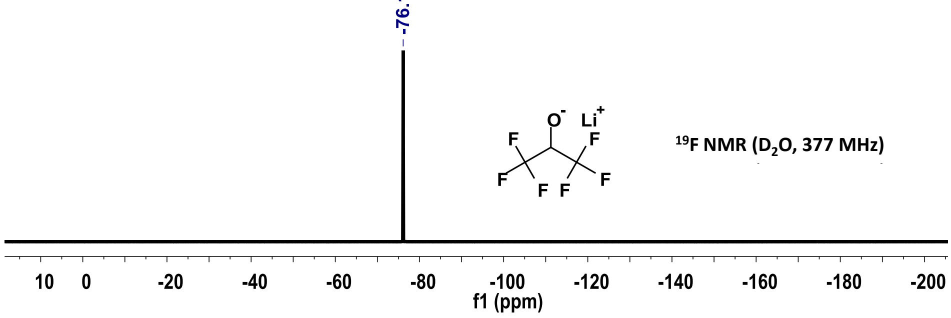

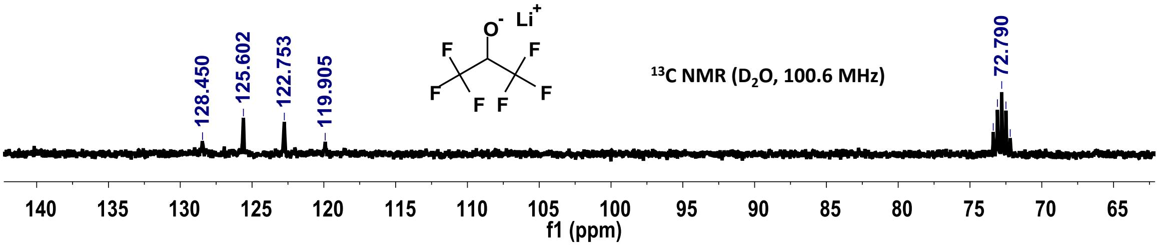

$LiOCH_2CF_3$

^1H NMR (D_2O, 400 MHz) δ 3.80-3.88 (m, 2H); ^{19}F NMR (D_2O, 377 MHz) δ -76.44; ^{13}C NMR (D_2O, 100.6 MHz) δ 131.0, 128.2, 125.4, 122.6, 62.2, 61.9, 61.6, 61.3.

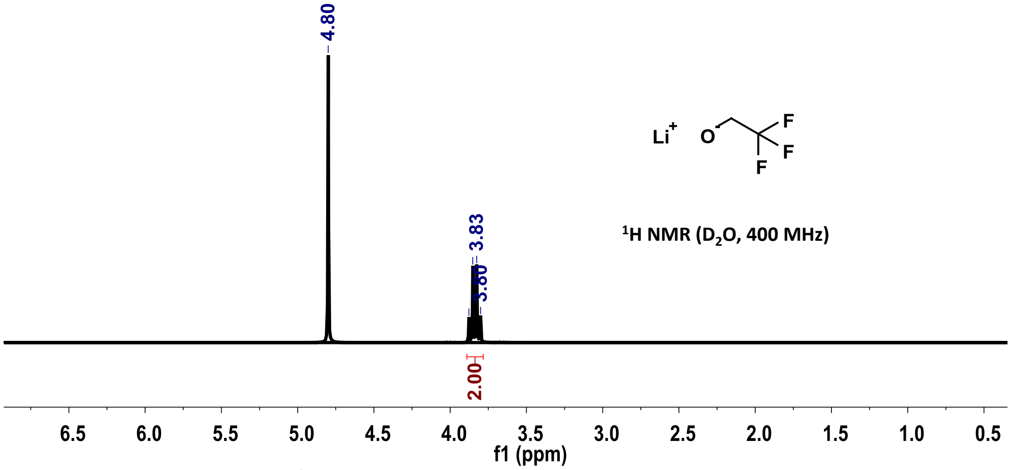

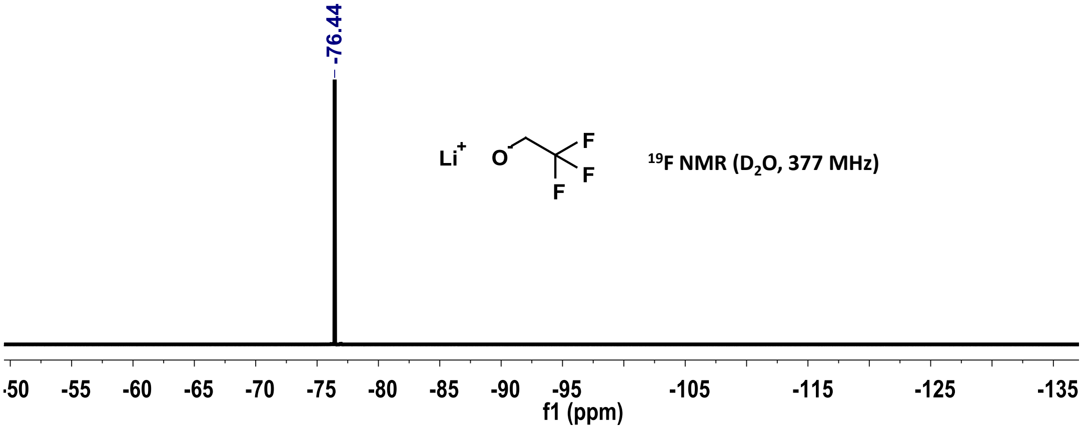

2. Coordination effect of different lithium salts with $PEG_{150}MeTMG$

Table S1 Coordination effect of different lithium salts with $PEG_{150}MeTMG$ [a]

Entry	Lithium salt	Coordination
1	LiOAc	No
2	LiCl	No
3	$LiClO_4$	No
4	Li_2WO_4	No
5	LiOH	No
6	$LiOCH_2CF_3$	No
7	$LiOCH(CF_3)_2$	No
8	$LiSO_3CF_3$	Yes
9	$LiNTf_2$	Yes

[a] The reaction was conducted by mixing $PEG_{150}MeTMG$ with the lithium salt in 1:1 molar ratio at room temperature. Homogeneous phases formed through multisite coordination interaction within 1 h. Otherwise, two separate phases were retained after stirring for 24 h.

3. ^{1}H NMR ($CDCl_3$, 400 MHz) spectrum of the neutral ligands and the corresponding chelate ionic liquids

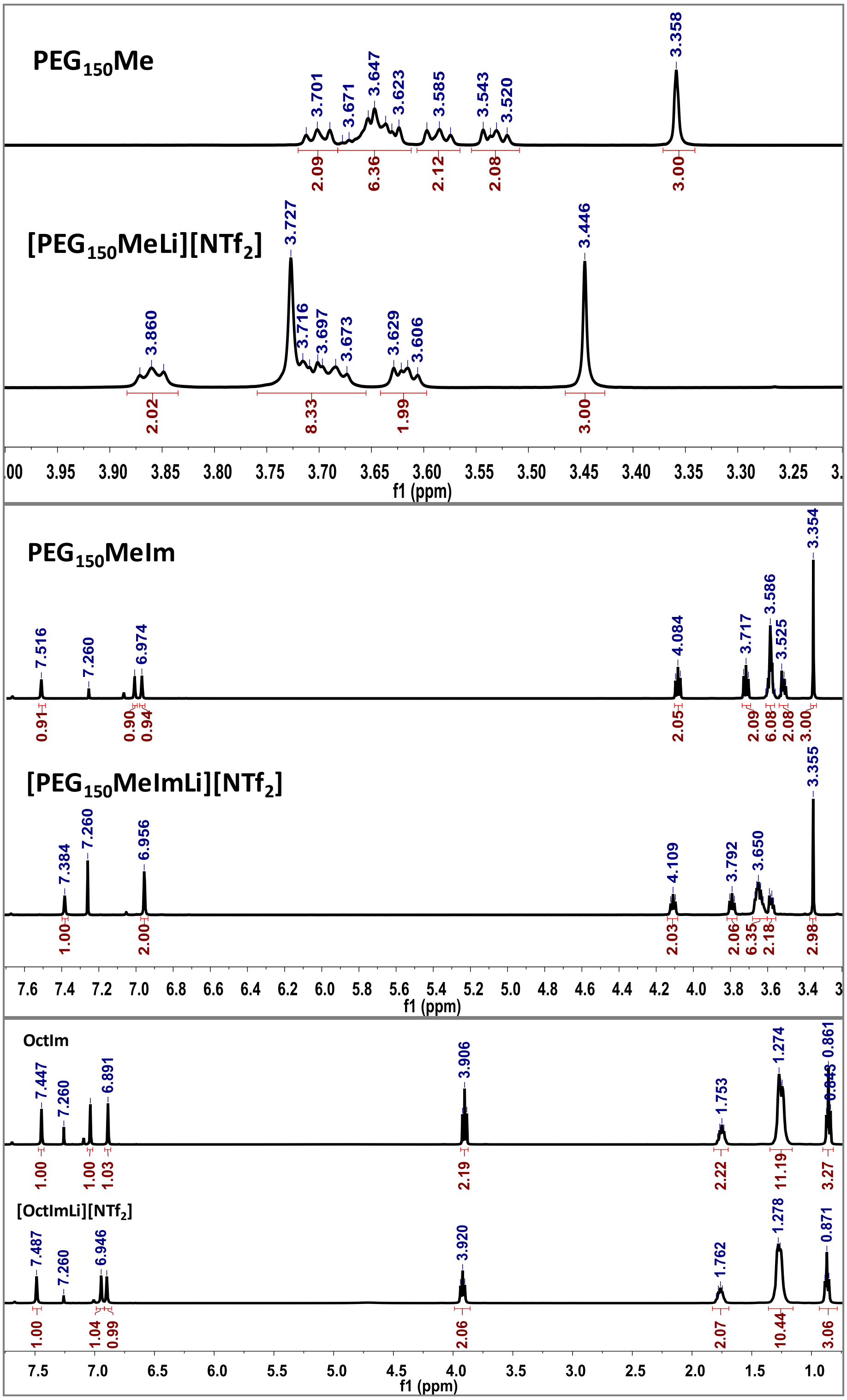

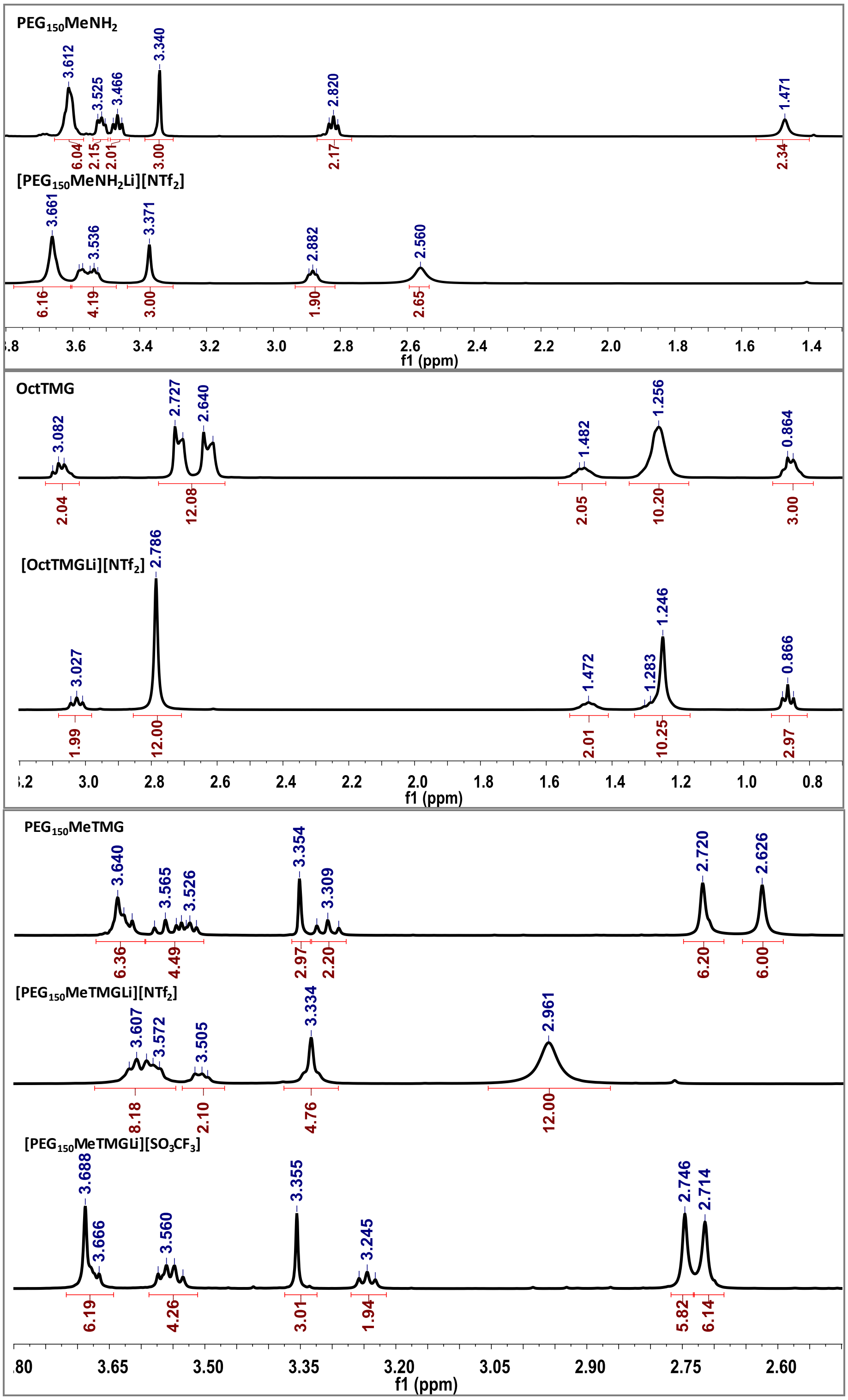
PEG150MeNH2
[PEG150MeNH2Li][NTf2]
f1 (ppm)
OctTMG
[OctTMGLi][NTf2]
f1 (ppm)
PEG150MeTMG
[PEG150MeTMGLi][NTf2]
[PEG150MeTMGLi][SO3CF3]
f1 (ppm)

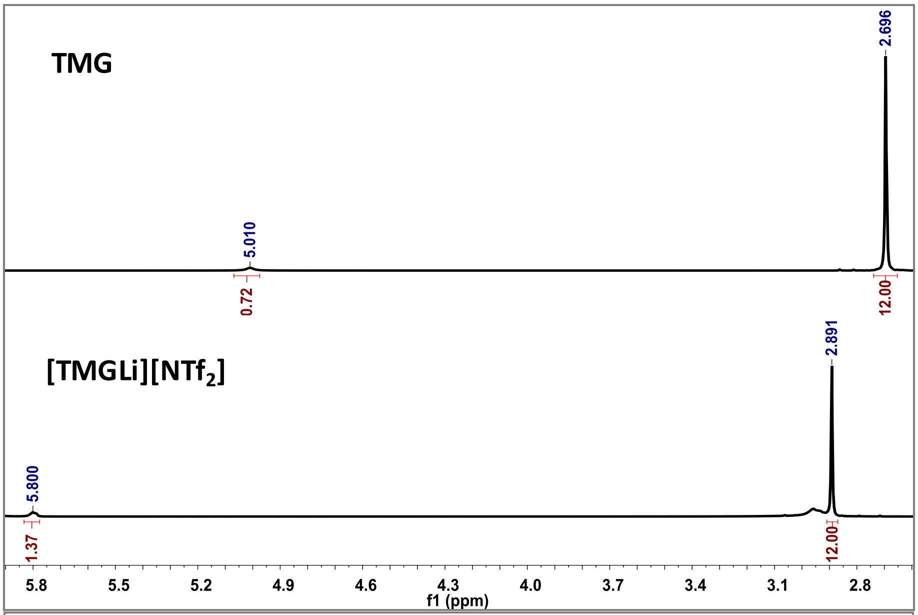

TMG
[TMGLi][NTf2]
f1 (ppm)

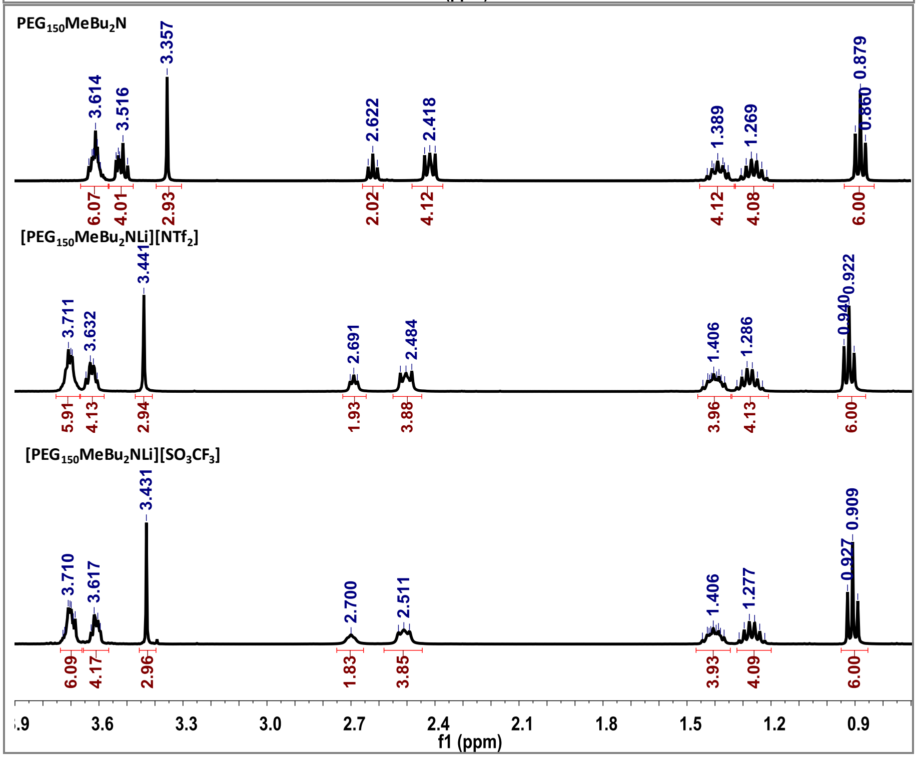

PEG150MeBu2N
[PEG150MeBu2NLi][NTf2]
[PEG150MeBu2NLi][SO3CF3]
f1 (ppm)

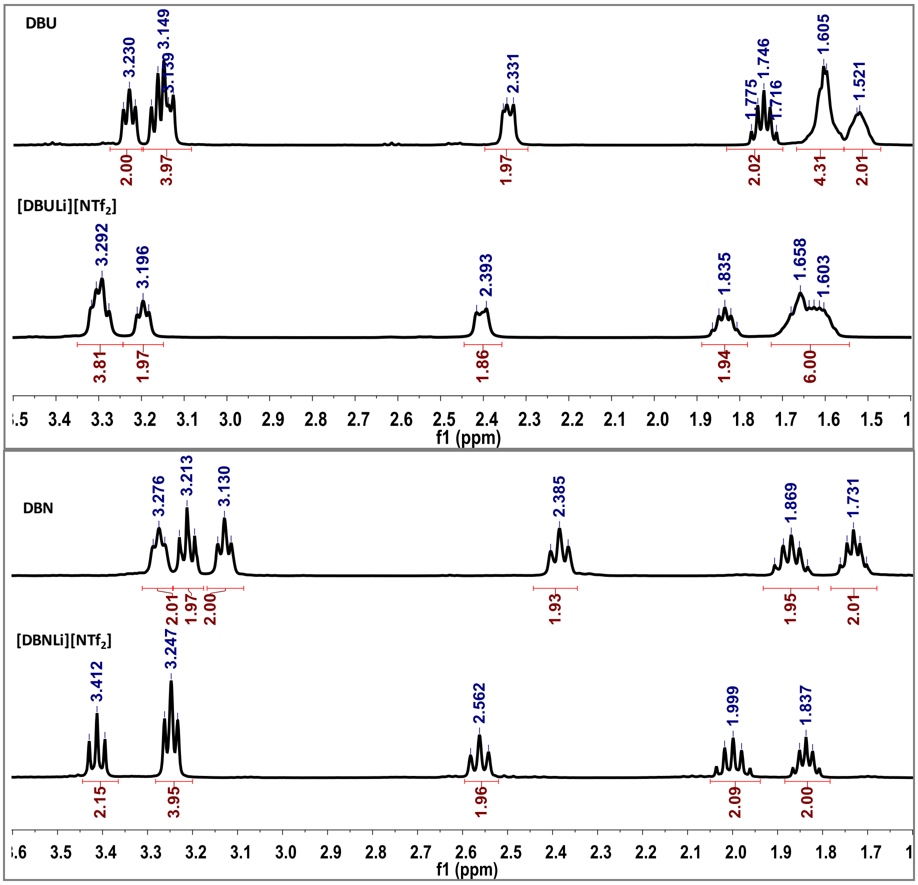

Figure S1 ^{1}H NMR ($CDCl_3$, 400 MHz) spectrum of the neutral ligands and the corresponding chelate ionic liquids after coordinated with lithium salts ($LiNTf_2$ and $LiSO_3CF_3$).

4. ESI spectrum of $PEG_{150}MeTMG$, $PEG_{150}MeBu_2N$ before and after coordinating with $LiNTf_2$

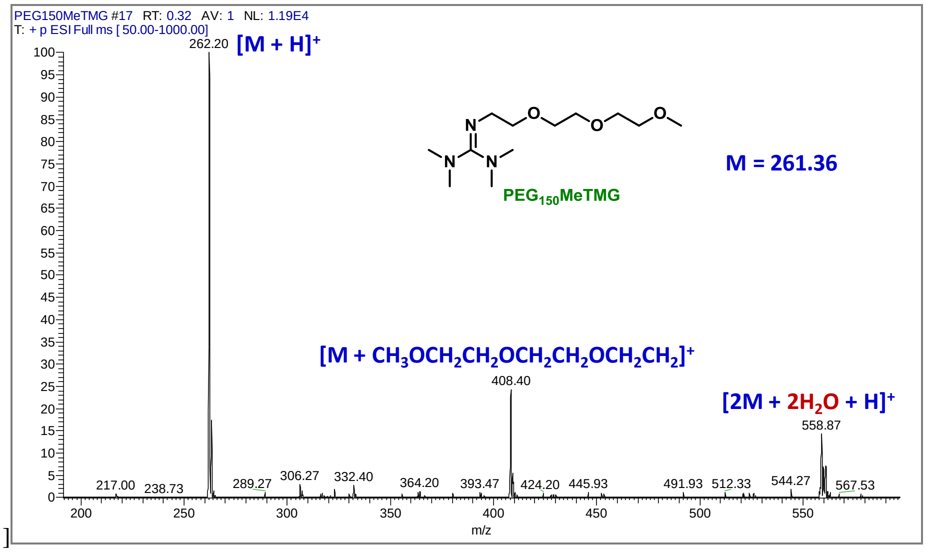

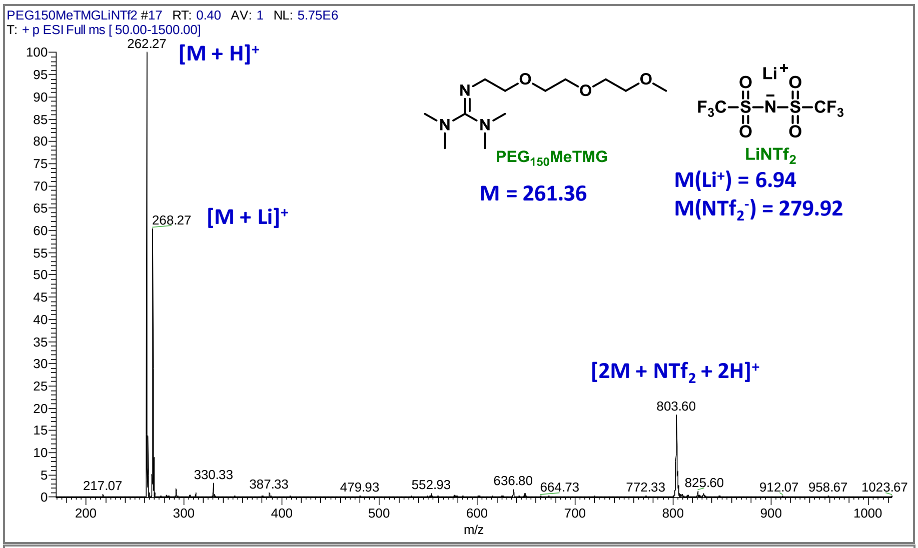

PEG150MeTMGLiNTf2 #17 RT: 0.40 AV: 1 NL: 5.75E6
T: + p ESI Full ms [50.00-1500.00]
[M + H]+
262.27
268.27
[M + Li]+
PEG150MeTMG
M = 261.36
LiNTf2
M(Li+) = 6.94
M(NTf2-) = 279.92
[2M + NTf2 + 2H]+
803.60
m/z

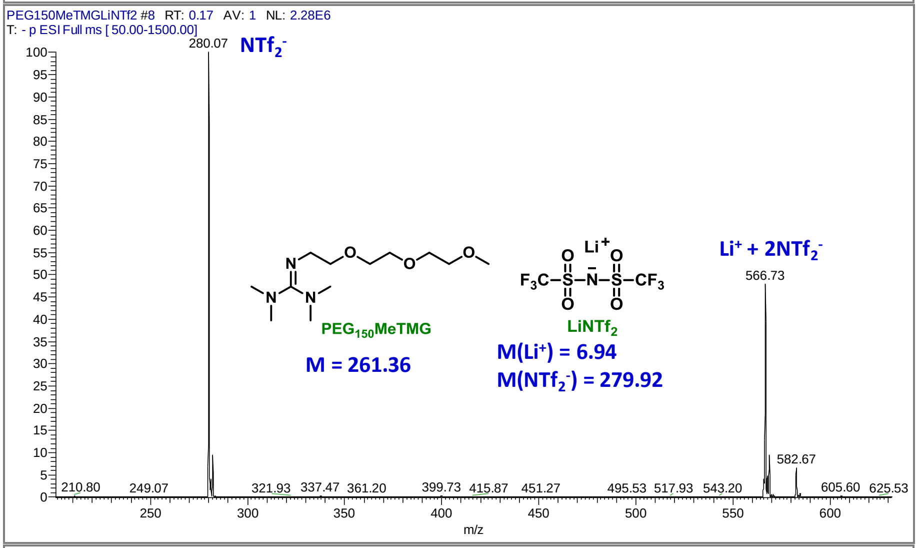

PEG150MeTMGLiNTf2 #8 RT: 0.17 AV: 1 NL: 2.28E6
T: - p ESI Full ms [50.00-1500.00]
280.07
NTf2-
PEG150MeTMG
M = 261.36
LiNTf2
M(Li+) = 6.94
M(NTf2-) = 279.92
Li+ + 2NTf2-
566.73
582.67
m/z

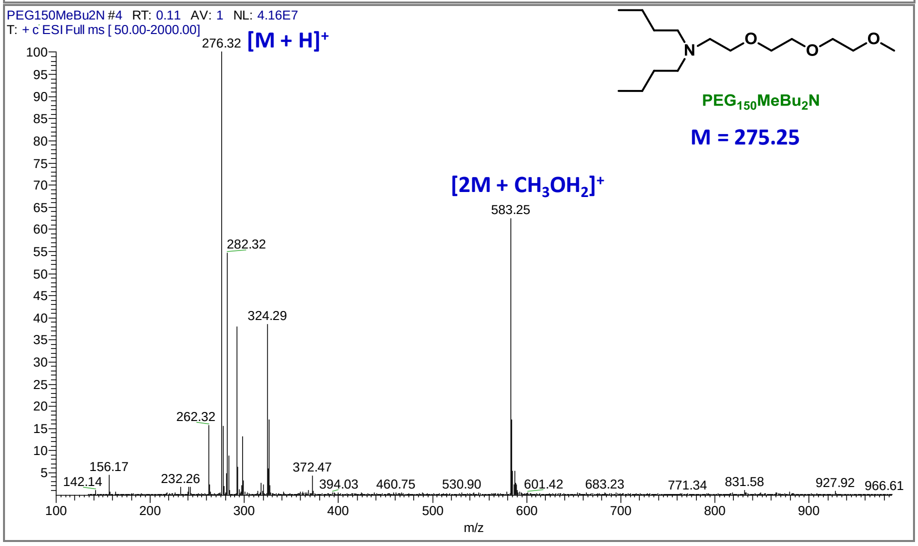

PEG150MeBu2N #4 RT: 0.11 AV: 1 NL: 4.16E7
T: + c ESI Full ms [50.00-2000.00]
276.32 [M + H]+
PEG150MeBu2N
M = 275.25
[2M + CH3OH2]+
583.25
282.32
324.29
262.32
m/z

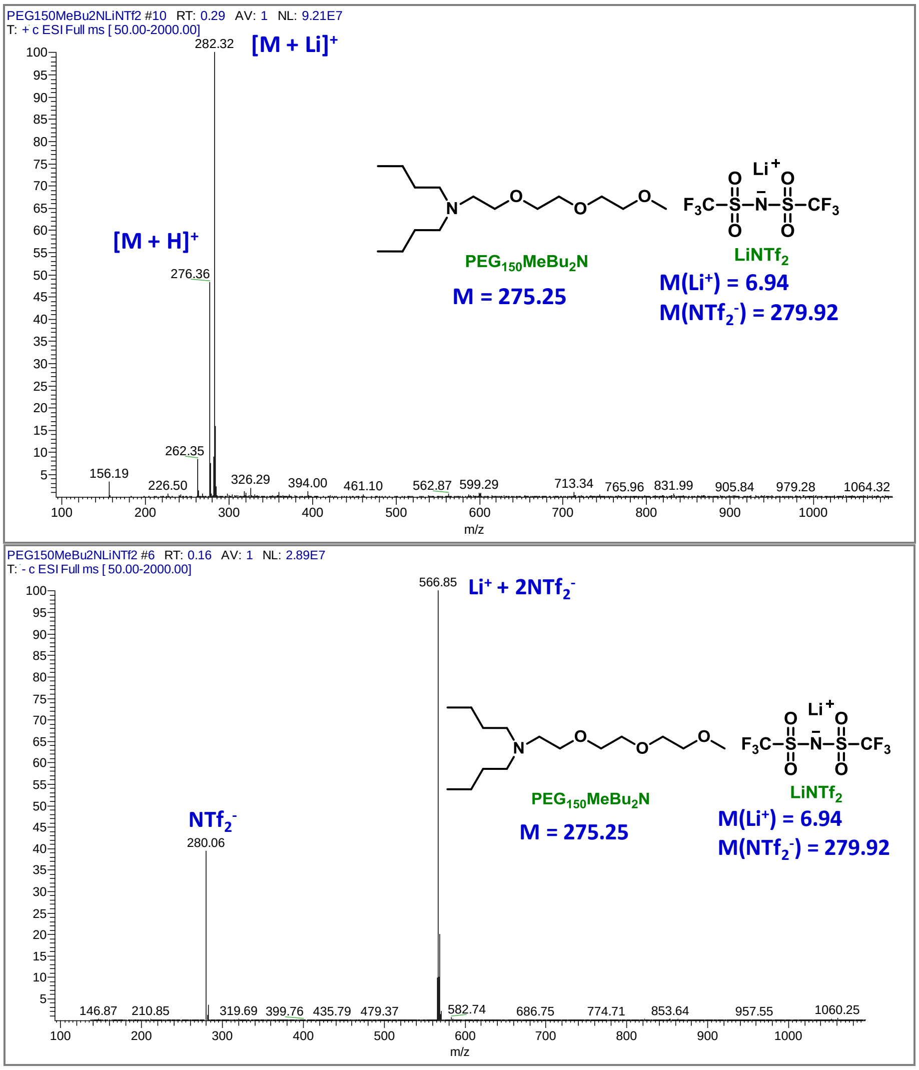

Figure S2 ESI spectrum of $PEG_{150}MeTMG$, $PEG_{150}MeBu_2N$ before and after coordinating with $LiNTf_2$.

5. References

1. Z.-Z. Yang, L.-N. He, Y.-N. Zhao and B. Yu, *Environ. Sci. Technol.*, **2013**, 47, 1598-1605.

2. H. Yang, Z. Ma, Y. Qing, G. Xie, J. Gao, L. zhang, J. Gao and L. du, *Appl. Catal. A-Gen.*, **2010**, 382, 312-321.

6. NMR characterization and data on all neutral ligands and chelate ionic liquids

$PEG_{150}Me$

^1H NMR ($CDCl_3$, 400 MHz) δ 3.69-3.71 (m, 2H), 3.62-3.67 (m, 6H), 3.57-3.60 (m, 2H), 3.52-3.54 (m, 2H), 3.36 (s, 3H); ^{13}C NMR ($CDCl_3$, 100.6 MHz) δ 72.4, 71.8, 70.5, 70.4, 70.3, 61.6, 59.0.

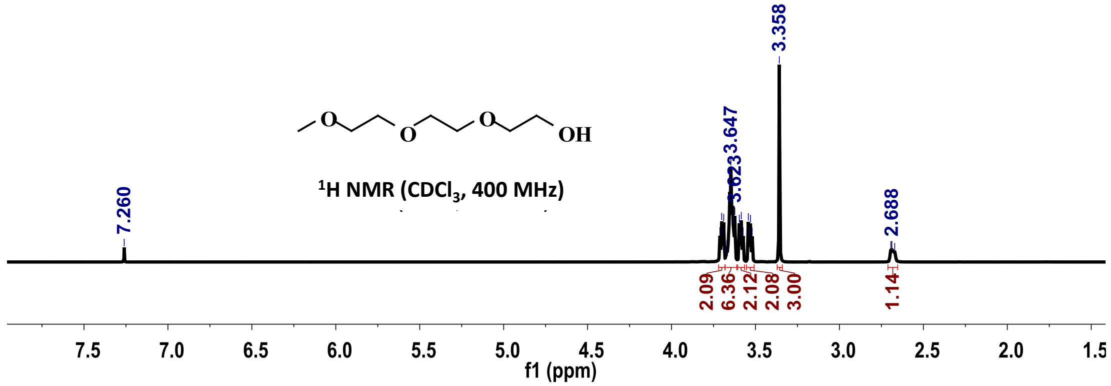

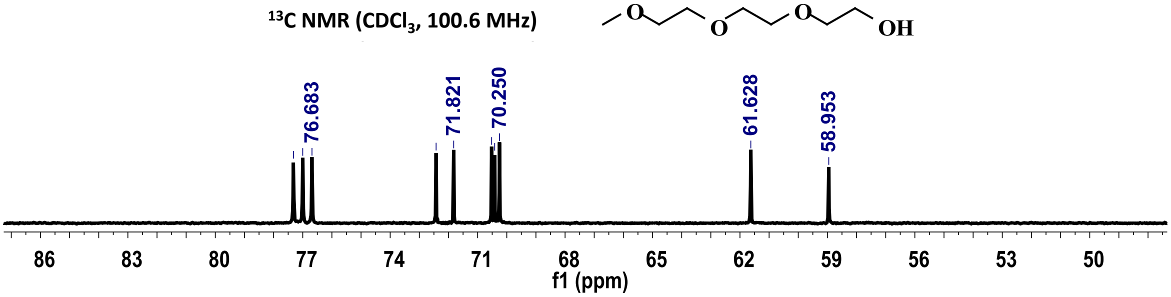

$PEG_{150}Me/LiNTf_2$

^{1}H NMR ($CDCl_3$, 400 MHz) δ 3.86 (t, ^{3}J = 4.4 Hz, 2H), 3.67-3.73 (m, 8H), 3.61-3.63 (m, 2H), 3.45 (s, 3H); ^{19}F NMR ($CDCl_3$, 377 MHz) δ -79.0; ^{13}C NMR ($CDCl_3$, 100.6 MHz) δ 124.3, 121.1, 117.9, 114.7, 70.1, 70.0, 68.3, 68.1, 67.7, 60.4, 58.9.

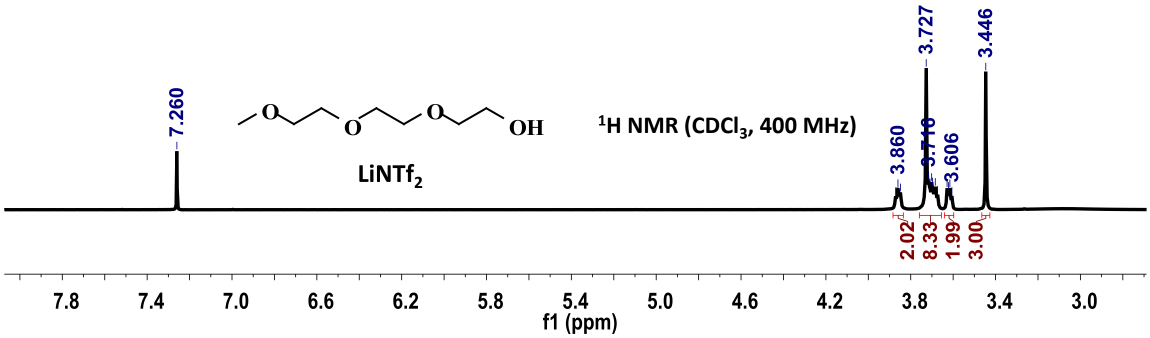

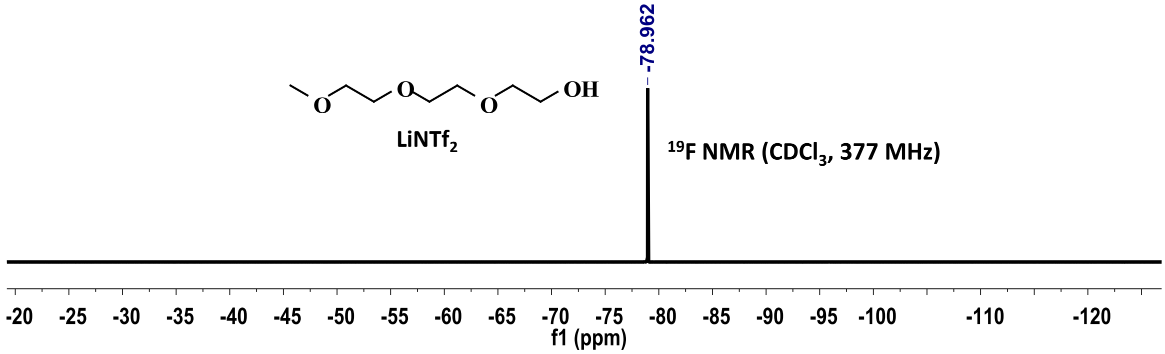

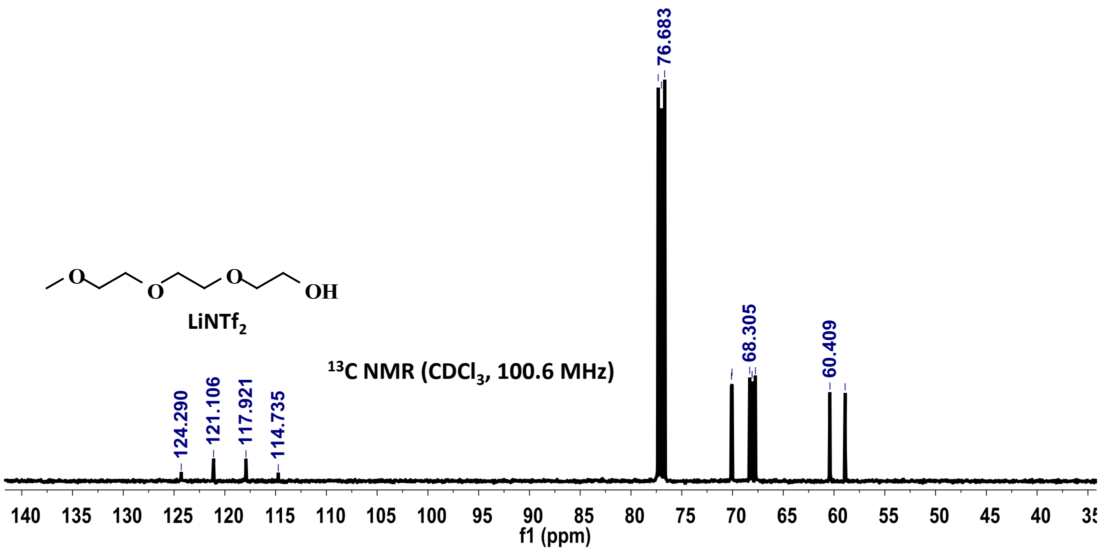

PEG$_{150}$MeIm

^{1}H NMR ($CDCl_3$, 400 MHz) δ 7.52 (s, 1H), 7.01 (s, 1H), 6.97 (s, 1H), 4.08 (t, 3J = 5.2 Hz, 2H), 3.72 (t, 3J = 5.2 Hz, 2H), 3.56-3.61 (m, 6H), 3.50-3.53 (m, 2H), 3.35 (s, 3H); ^{13}C NMR ($CDCl_3$, 100.6 MHz) δ 137.4, 129.0, 119.3, 71.8, 70.5, 70.4, 70.3, 58.9, 46.9. ESI-MS calcd for $C_{10}H_{18}N_2O_3$ 214.13, found 215.3 $[M+H]^+$.

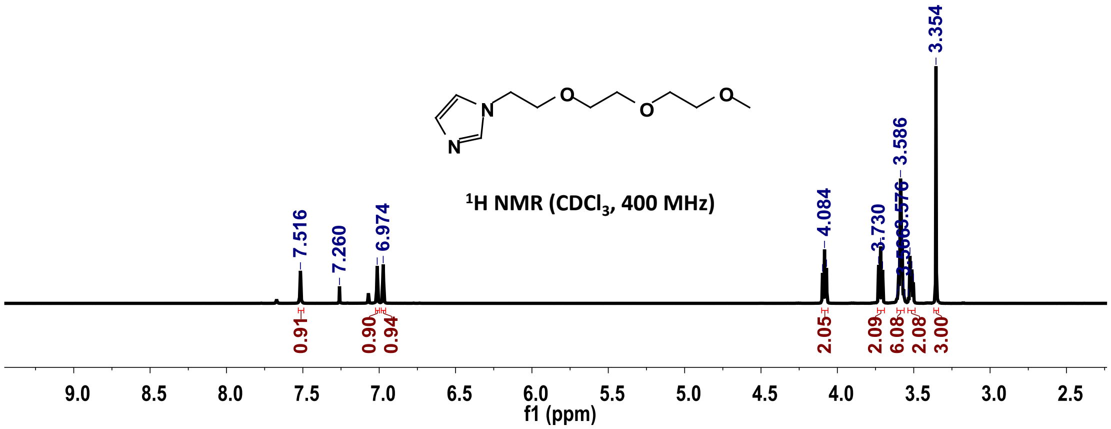

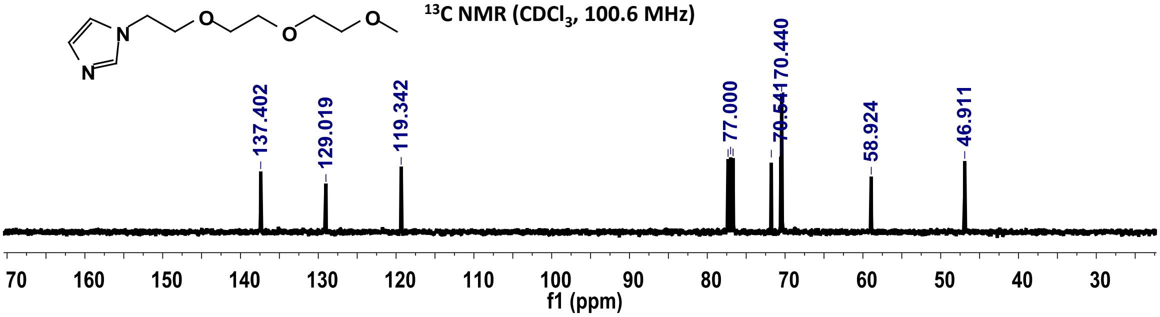

PEG$_{150}$MeIm/LiNTf$_2$

^{1}H NMR ($CDCl_3$, 400 MHz) δ 7.38 (s, 1H), 6.96 (s, 2H), 4.11 (t, ^3J = 5.2 Hz, 2H), 3.79 (t, ^3J = 5.2 Hz, 2H), 3.62-3.67 (m, 6H), 3.57-3.59 (m, 2H), 3.35 (s, 3H); ^{19}F NMR ($CDCl_3$, 377 MHz) δ -78.9; ^{13}C NMR ($CDCl_3$, 100.6 MHz) δ 137.8, 128.4, 124.3, 121.2, 119.5, 117.9, 114.8, 70.9, 70.2, 69.8, 69.6, 69.1, 58.9, 46.7.

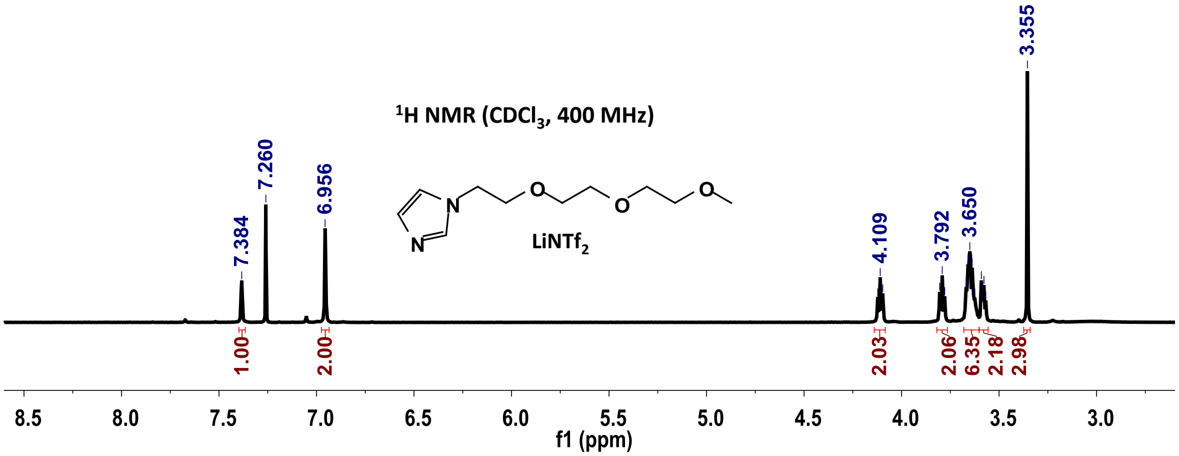

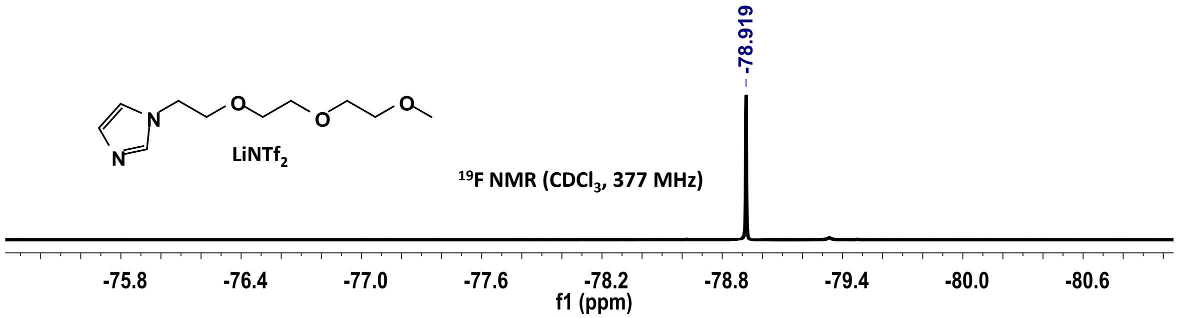

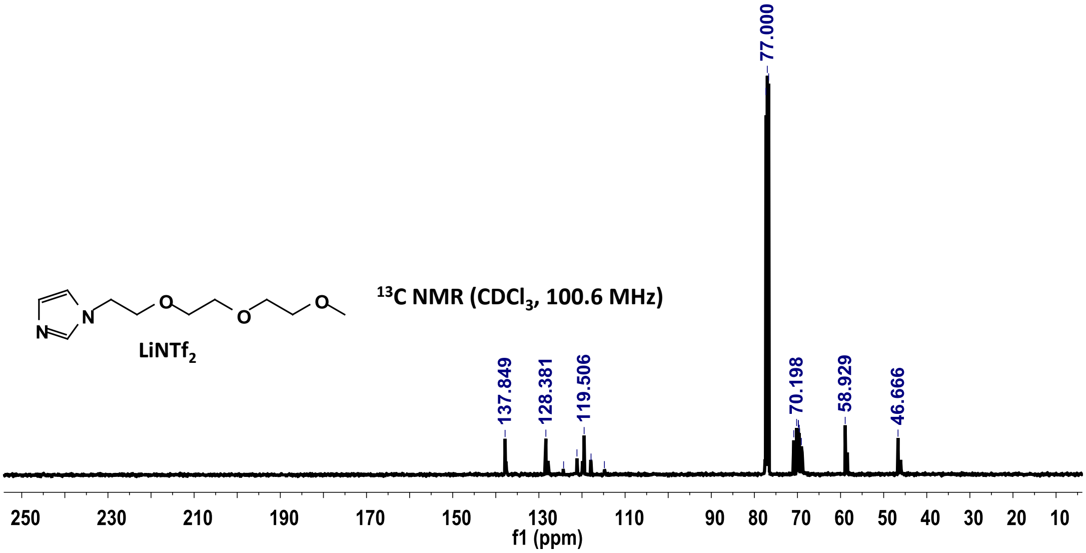

OctIm/LiNTf$_2$

^{1}H NMR (CDCl$_3$, 400 MHz) δ 7.49 (s, 1H), 6.95 (s, 1H), 6.90 (s, 1H), 3.92 (t, ^3J = 7.2 Hz, 2H), 1.75-1.80 (m, 2H), 1.26-1.28 (m, 10H), 0.87 (t, ^3J = 6.4 Hz, 3H); ^{19}F NMR (CDCl$_3$, 377 MHz) δ -79.2; ^{13}C NMR (CDCl$_3$, 100.6 MHz) δ 137.3, 128.3, 124.1, 120.9, 119.1, 117.7, 114.5, 47.4, 31.7, 30.8, 29.0, 28.9, 26.4, 22.5, 14.0.

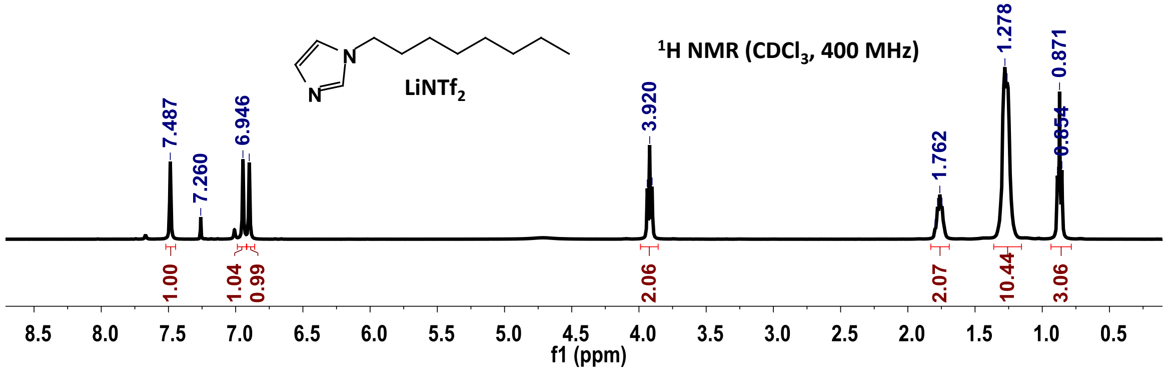

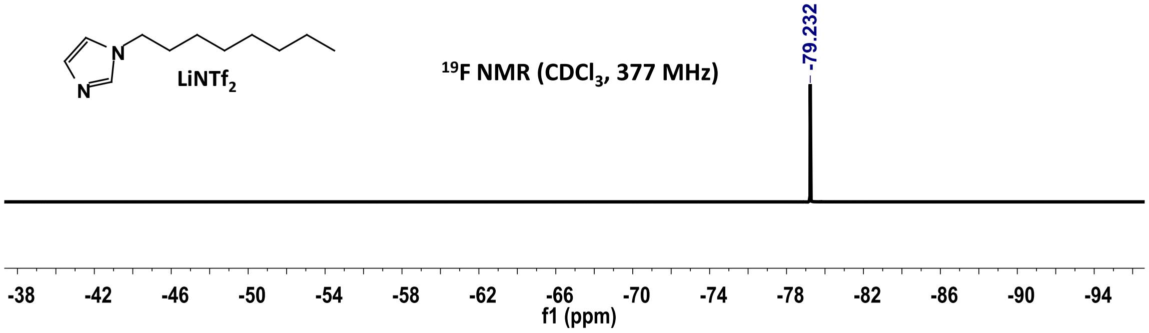

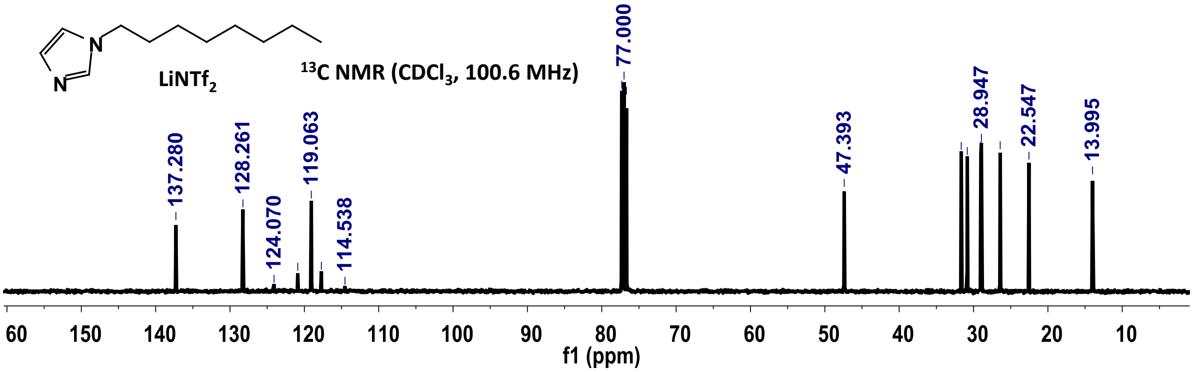

$PEG_{150}MeNH_2$

^{1}H NMR ($CDCl_3$, 400 MHz) δ 3.61 (s, 6H), 3.51 (t, ^{3}J = 4 Hz, 2H), 3.47 (t, ^{3}J = 4 Hz, 2H), 3.34 (s, 3H), 2.82 (t, ^{3}J = 4 Hz, 2H), 1.47 (s, 2H); ^{13}C NMR ($CDCl_3$, 100.6 MHz) δ 73.4, 71.8, 70.5, 70.4, 70.2, 58.9, 41.7.

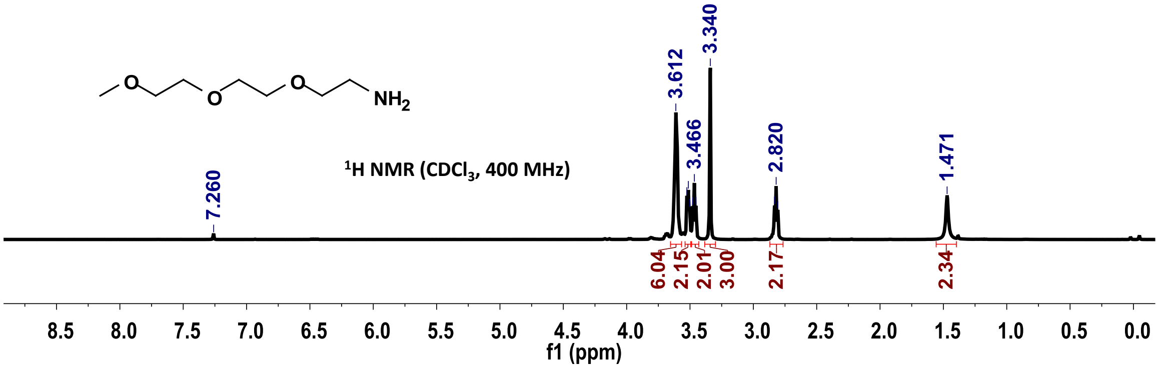

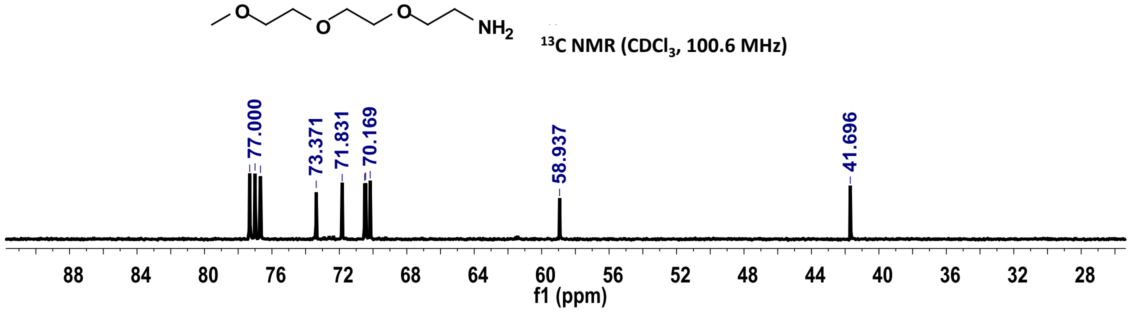

PEG$_{150}$MeNH$_2$/LiNTf$_2$

^{1}H NMR (CDCl$_3$, 400 MHz) δ 3.66 (s, 6H), 3.53-3.58 (m, 4H), 3.37 (s, 3H), 2.88 (t, ^3J = 4 Hz, 2H), 2.56 (s, 4H); ^{19}F NMR (CDCl$_3$, 377 MHz) δ -78.9; ^{13}C NMR (CDCl$_3$, 100.6 MHz) δ 124.4, 121.2, 118.0, 114.8, 70.4, 70.3, 68.5, 68.3, 67.9, 58.7, 40.2.

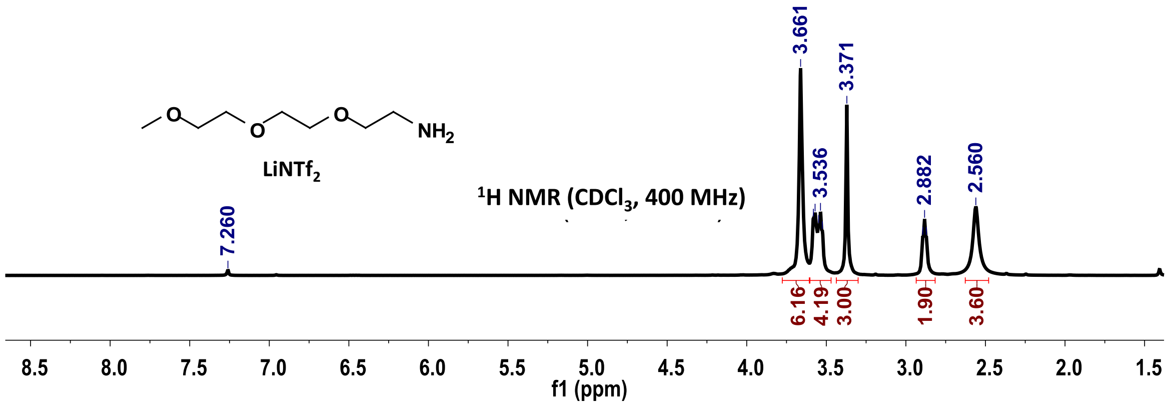

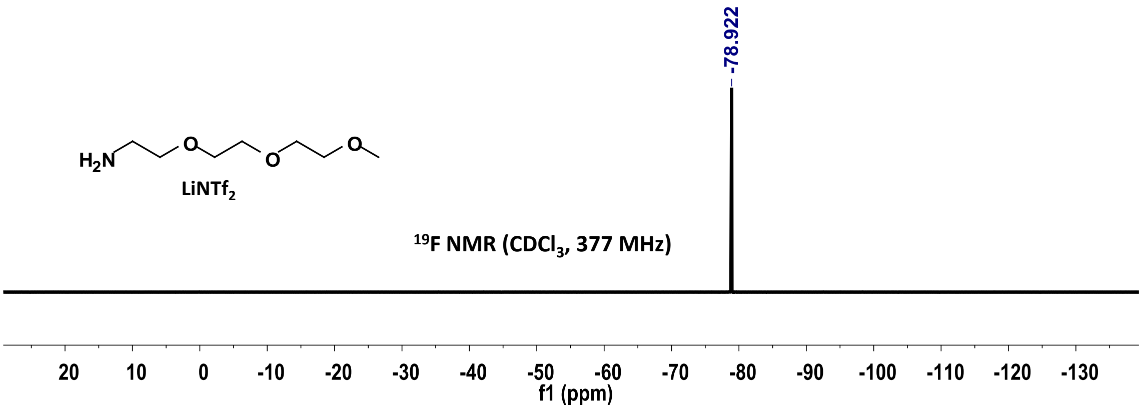

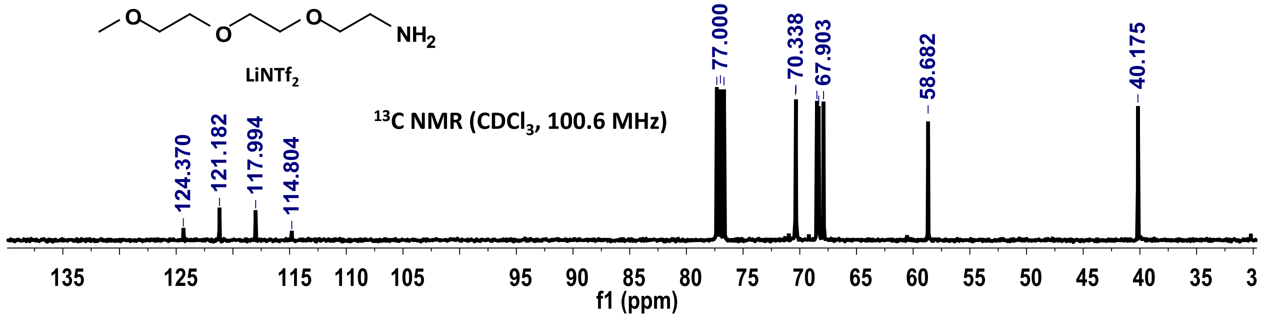

TMG

^{1}H NMR (CDCl$_3$, 400 MHz) δ 5.01 (s, 1H), 2.70 (s, 12H); ^{13}C NMR (CDCl$_3$, 100.6 MHz) δ 167.9, 39.3.

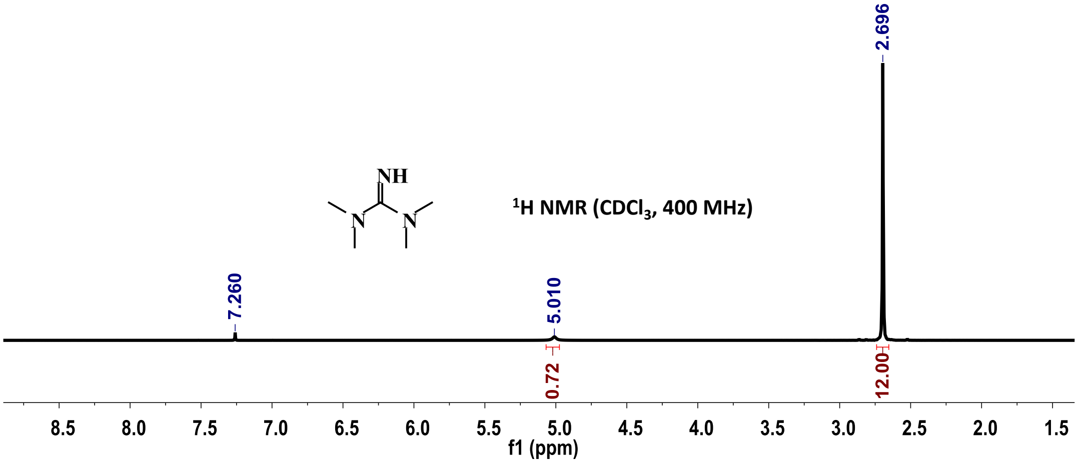

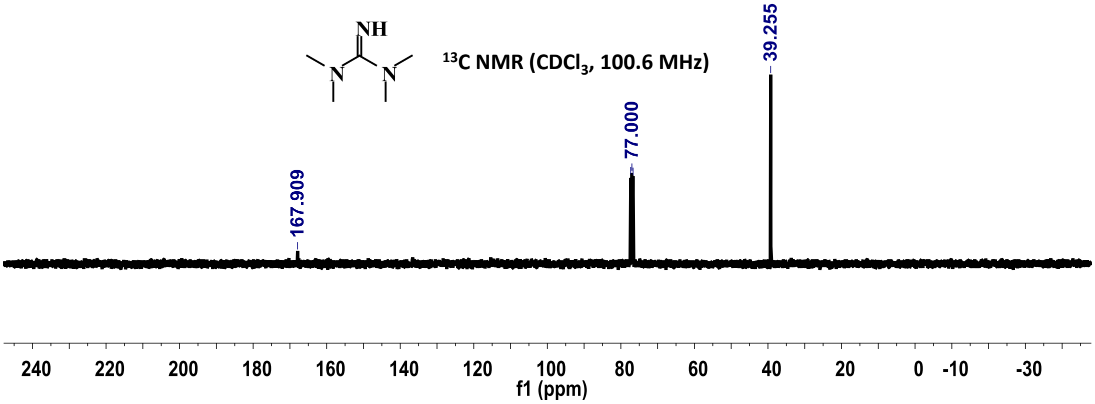

TMG/$LiNTf_2$

^{1}H NMR ($CDCl_3$, 400 MHz) δ 5.80 (s, 1H), 2.89 (s, 12H); ^{19}F NMR ($CDCl_3$, 377 MHz) δ -79.0; ^{13}C NMR ($CDCl_3$, 100.6 MHz) δ 163.9, 124.4, 121.2, 118.0, 114.8, 39.4.

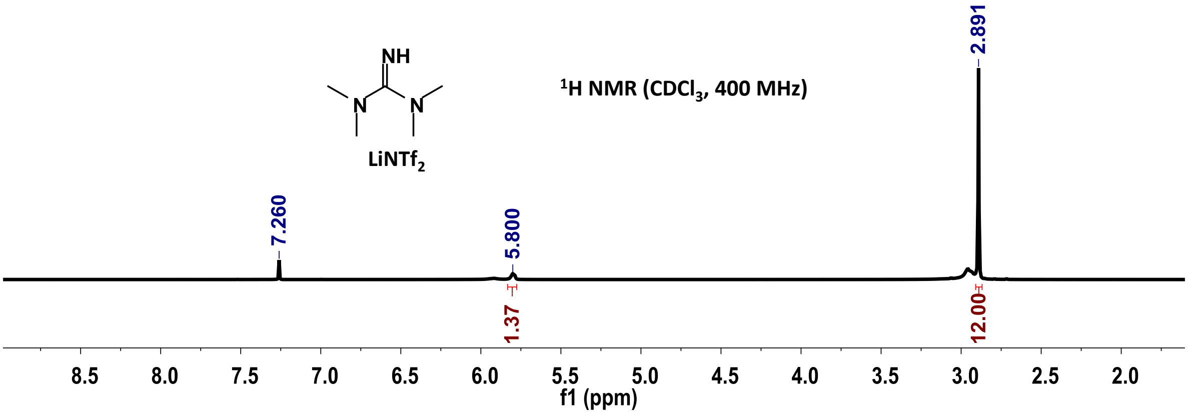

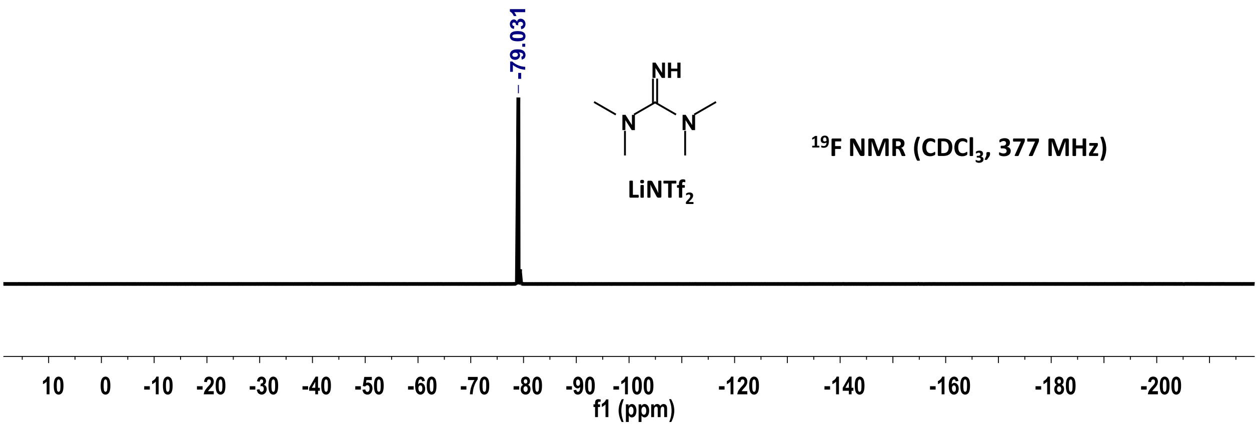

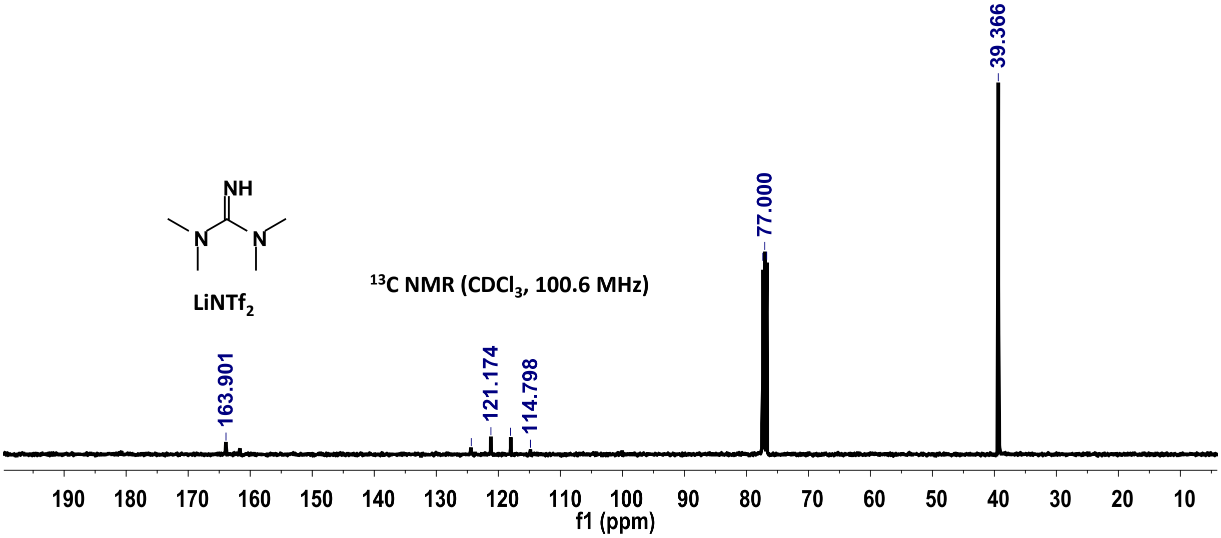

OctTMG

^{1}H NMR ($CDCl_3$, 400 MHz) δ 3.07-3.10 (m, 2H), 2.61-2.73 (m, 12H), 1.48-1.50 (m, 2H), 1.26 (m, 10H), 0.85-0.86 (m, 3H); ^{13}C NMR ($CDCl_3$, 100.6 MHz) δ 159.8, 49.6, 39.6, 38.8, 32.8, 31.9, 29.5, 29.4, 27.5, 22.7, 14.1.

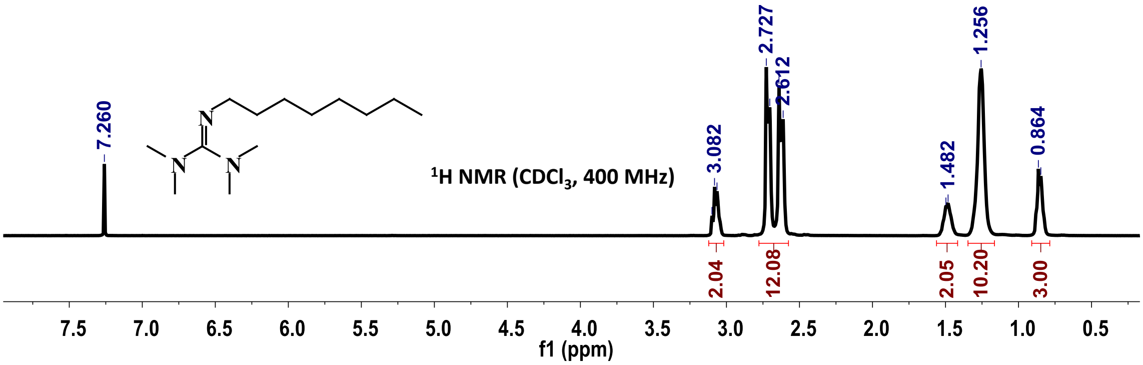

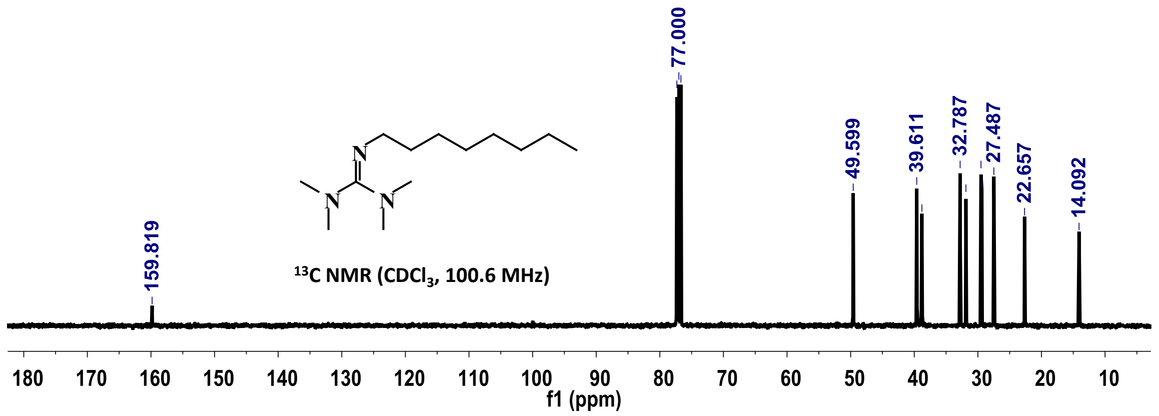

OctTMG/LiNTf$_2$

^{1}H NMR ($CDCl_3$, 400 MHz) δ 3.03 (t, ^{3}J = 6.8 Hz, 2H), 2.79 (s, 12H), 1.47 (s, 2H), 1.25-1.30 (m, 10H), 0.87 (t, ^{3}J = 6.4 Hz, 3H); ^{19}F NMR ($CDCl_3$, 377 MHz) δ -78.9; ^{13}C NMR ($CDCl_3$, 100.6 MHz) δ 162.6, 124.0, 120.9, 117.7, 114.5, 47.9, 39.6, 38.7, 31.8, 31.4, 29.22, 29.17, 27.0, 22.6, 14.0.

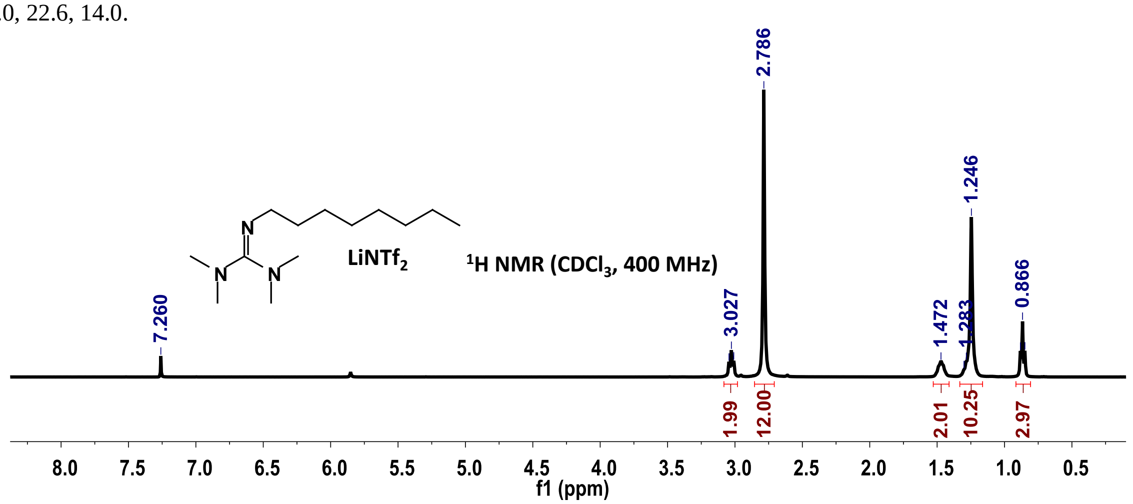

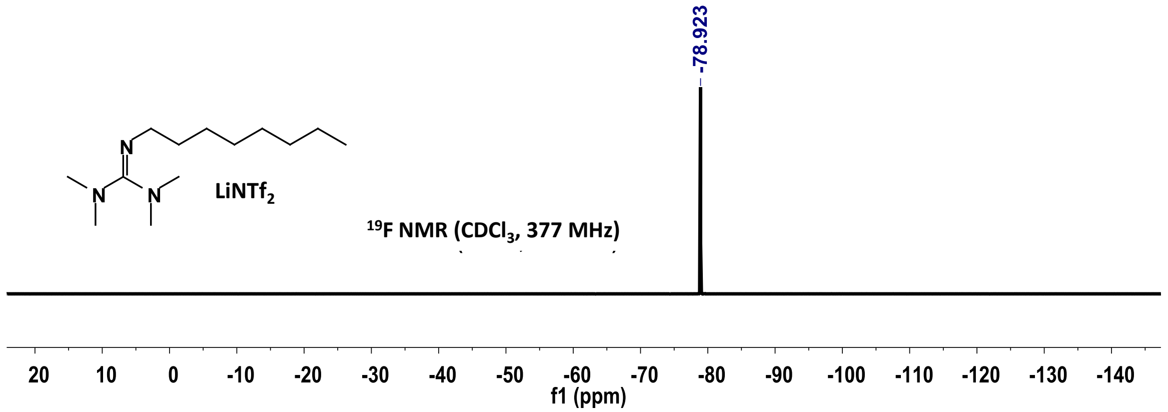

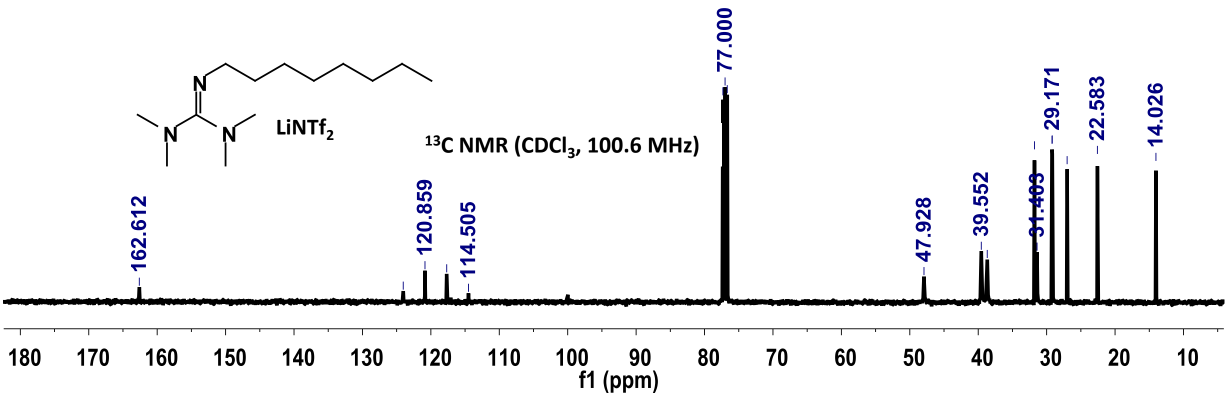

PEG$_{150}$MeTMG

^{1}H NMR ($CDCl_3$, 400 MHz) δ 3.52-3.64 (m, 10H), 3.35 (s, 3H), 3.31 (t, ^{3}J = 8 Hz, 2H), 2.72 (s, 6H), 2.63 (s, 6H); ^{13}C NMR ($CDCl_3$, 100.6 MHz) δ 160.9, 73.4, 71.9, 70.6, 70.4, 70.3, 59.0, 49.4, 39.6, 38.8.

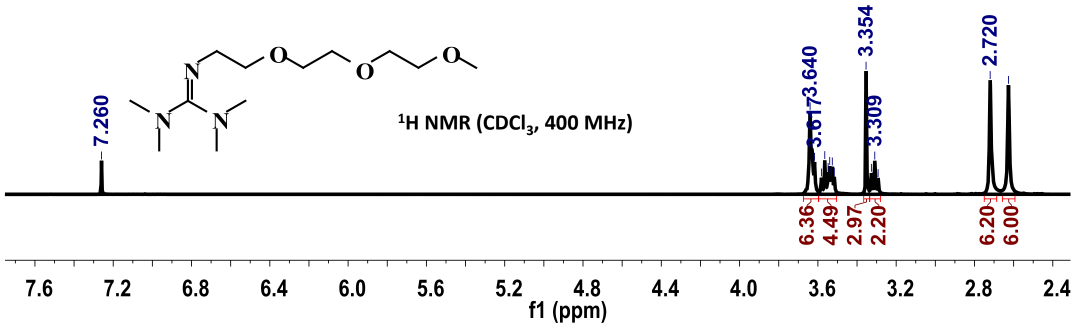

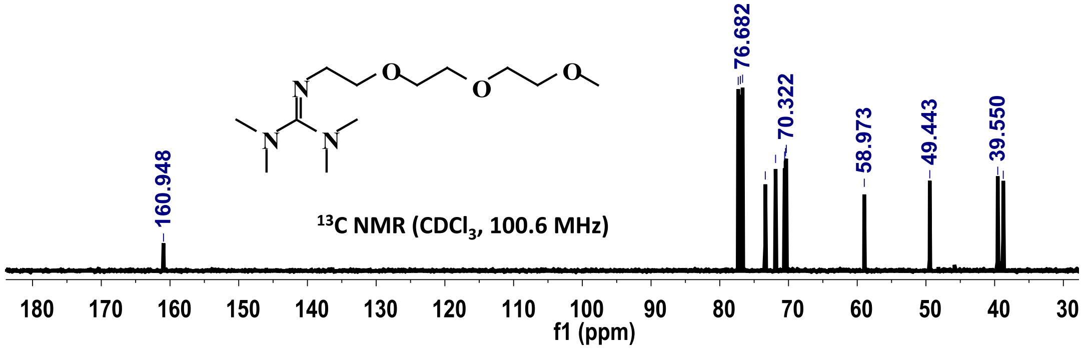

PEG$_{150}$MeTMG/LiNTf$_2$

^{1}H NMR ($CDCl_3$, 400 MHz) δ 3.57-3.62 (m, 8H), 3.50-3.52 (m, 2H), 3.33 (s, 5H), 2.96 (s, 12H); ^{19}F NMR ($CDCl_3$, 377 MHz) δ -78.9; ^{13}C NMR ($CDCl_3$, 100.6 MHz) δ 162.4, 124.5, 121.4, 118.2, 115.0, 71.7, 70.2, 70.1, 69.3, 58.8, 45.0, 39.7.

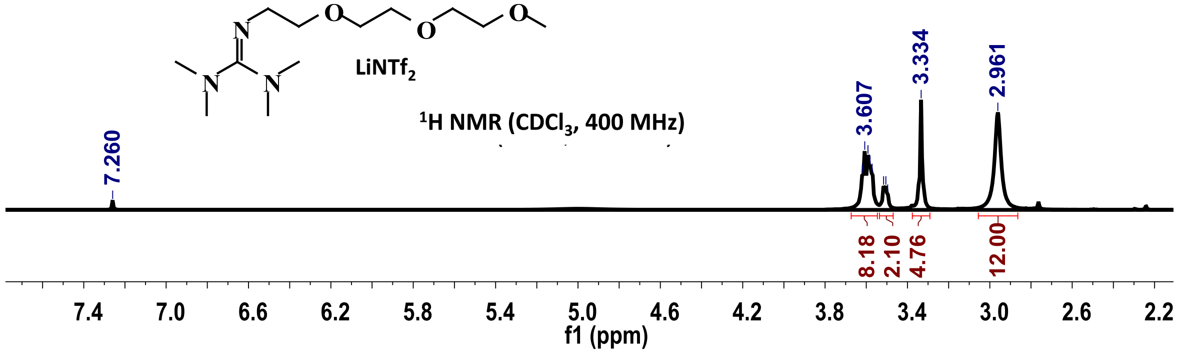

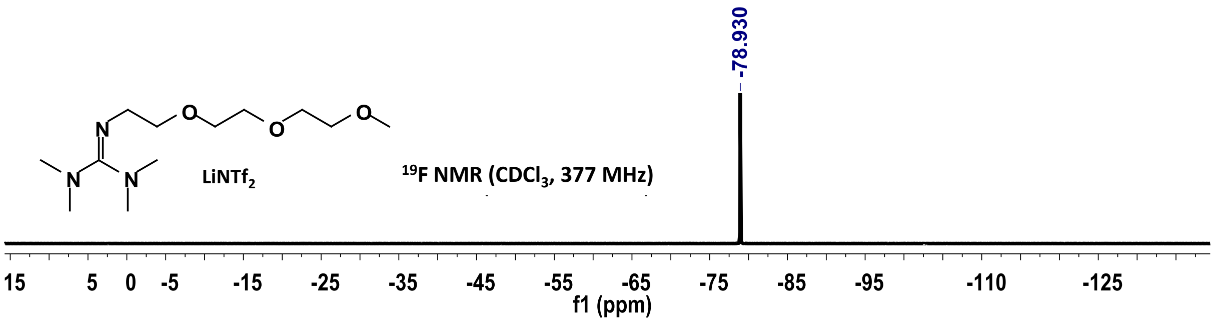

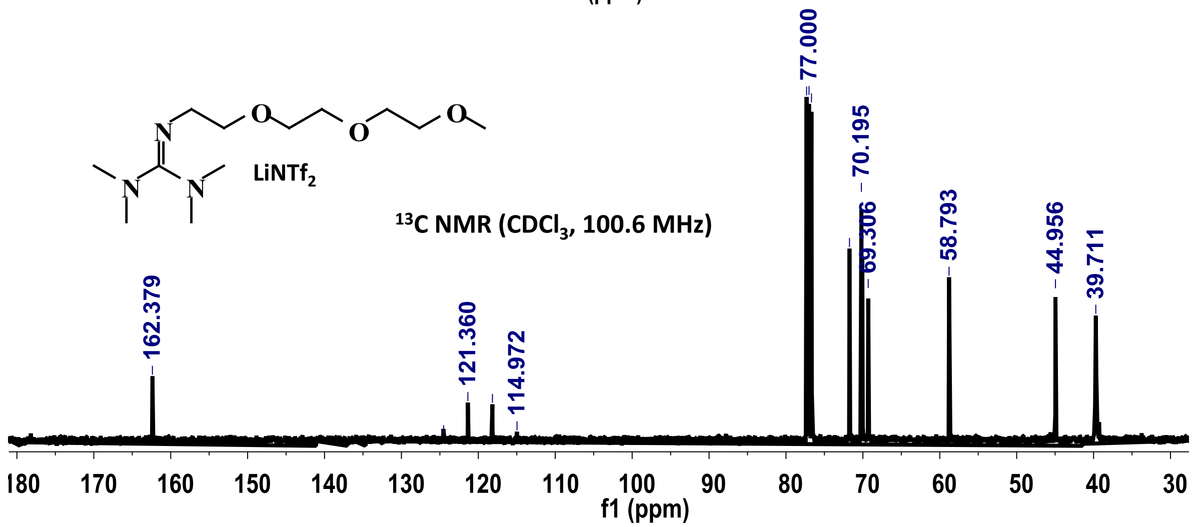

$PEG_{150}MeTMG/LiSO_3CF_3$

^{1}H NMR ($CDCl_3$, 400 MHz) δ 3.67-3.69 (m, 6H), 3.53-3.57 (m, 4H), 3.36 (s, 3H), 3.24 (t, ^3J = 5.2 Hz, 2H), 2.75 (s, 6H), 2.71 (s, 6H); ^{19}F NMR ($CDCl_3$, 377 MHz) δ -78.4; ^{13}C NMR ($CDCl_3$, 100.6 MHz) δ 163.1, 125.0, 121.8, 118.6, 115.5, 72.4, 70.8, 69.2, 58.8, 47.9, 39.5, 38.6.

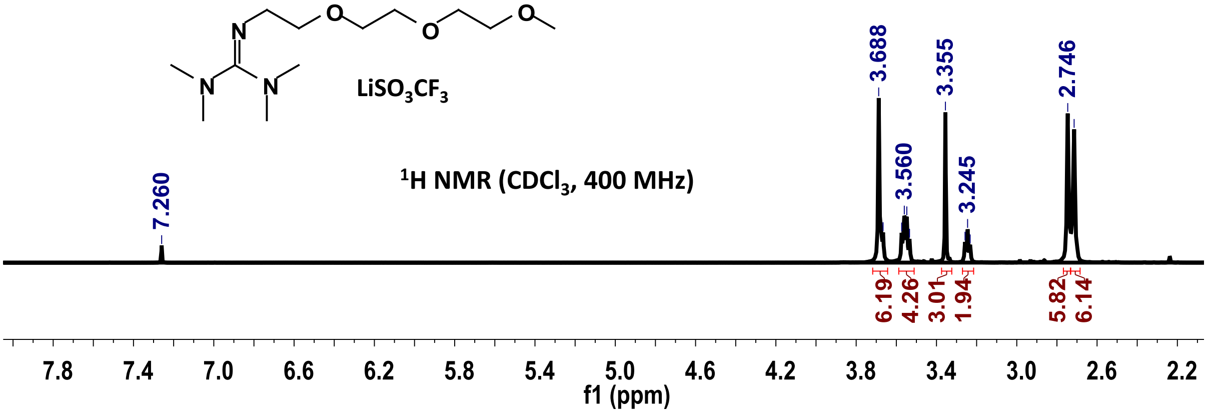

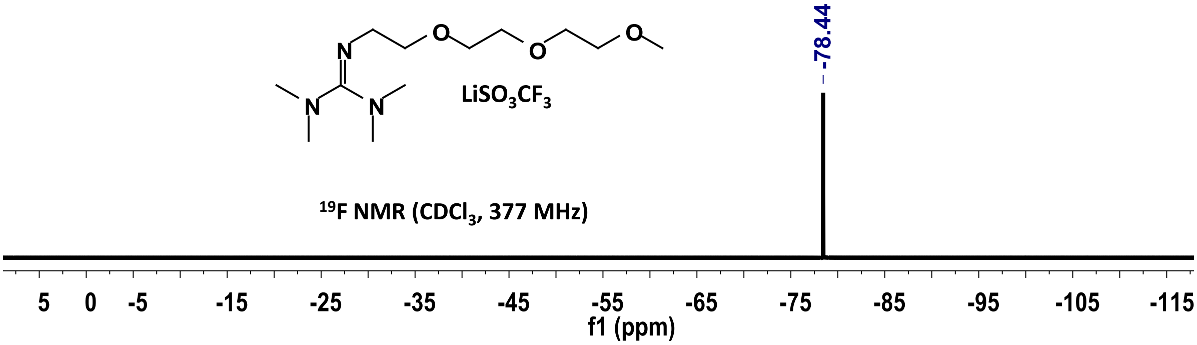

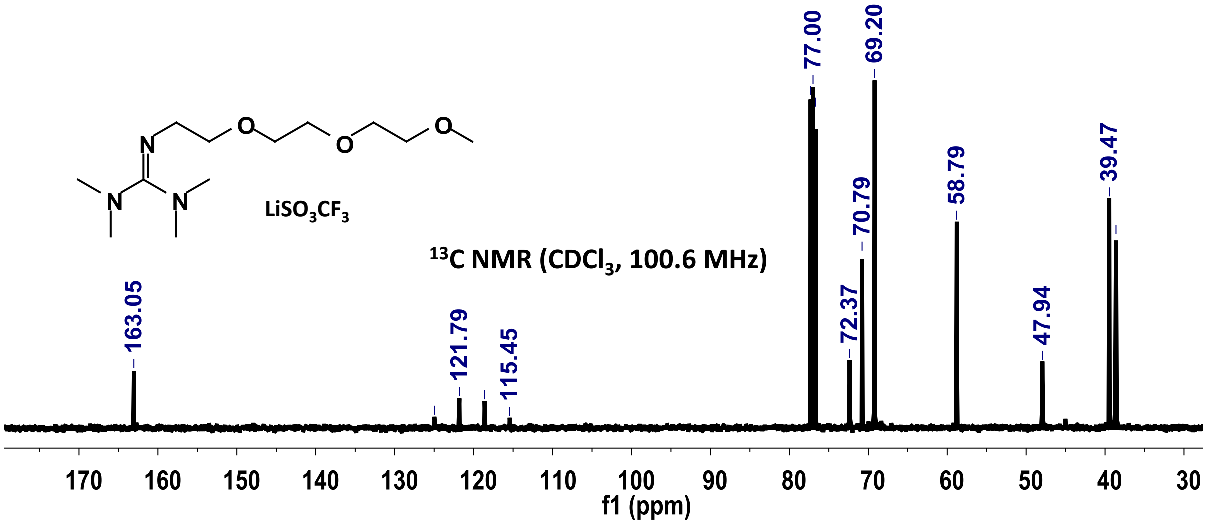

OctBu$_2$N

^{1}H NMR ($CDCl_3$, 400 MHz) δ 2.35-2.39 (m, 6H), 1.36-1.43 (m, 6H), 1.26-1.33 (m, 14H), 0.85-0.92 (m, 9H); ^{13}C NMR ($CDCl_3$, 100.6 MHz) δ 54.3, 53.9, 31.9, 29.6, 29.3, 29.2, 27.7, 27.1, 22.7, 20.8, 14.1.

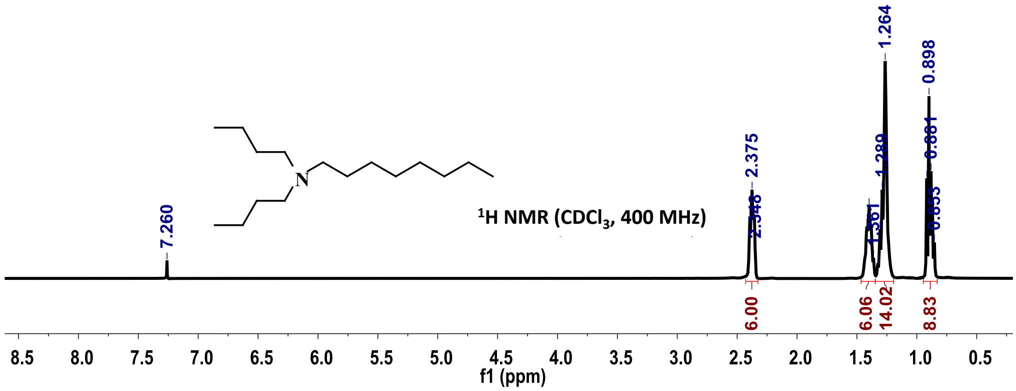

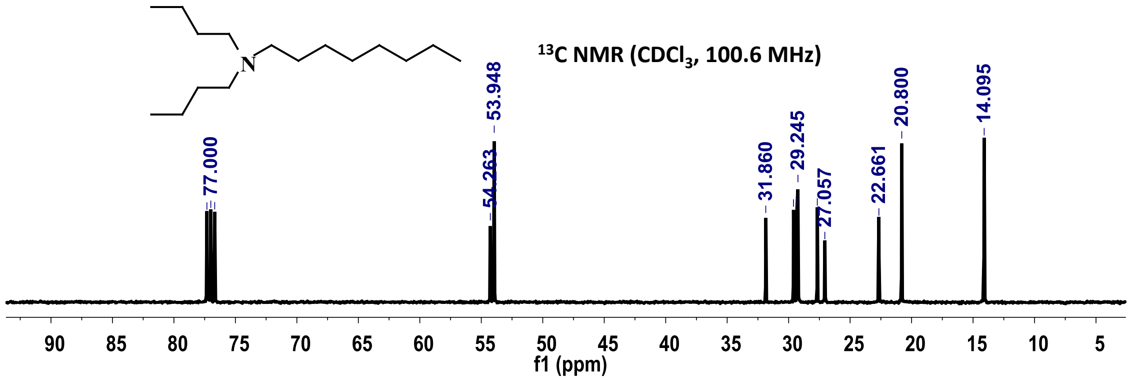

PEG$_{150}$MeBu$_2$N

^{1}H NMR ($CDCl_3$, 400 MHz) δ 3.60-3.64 (m, 6H), 3.50-3.54 (m, 4H), 3.36 (s, 3H), 2.62 (t, ^3J = 6.8 Hz, 2H), 2.42 (t, ^3J = 7.6 Hz, 4H), 1.35-1.43 (m, 4H), 1.21-1.30 (m, 4H), 0.88 (t, ^3J = 7.2 Hz, 6H); ^{13}C NMR ($CDCl_3$, 100.6 MHz) δ 71.9, 70.6, 70.5, 70.4, 69.9, 59.0, 54.6, 53.4, 29.2, 20.6, 14.0.

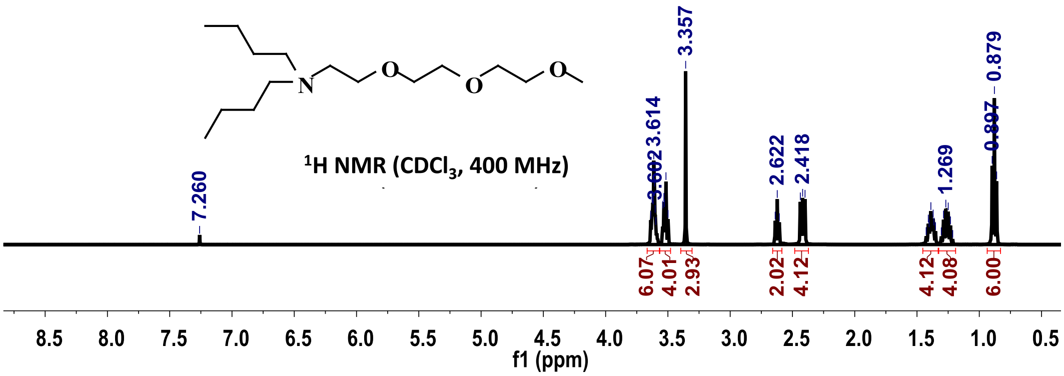

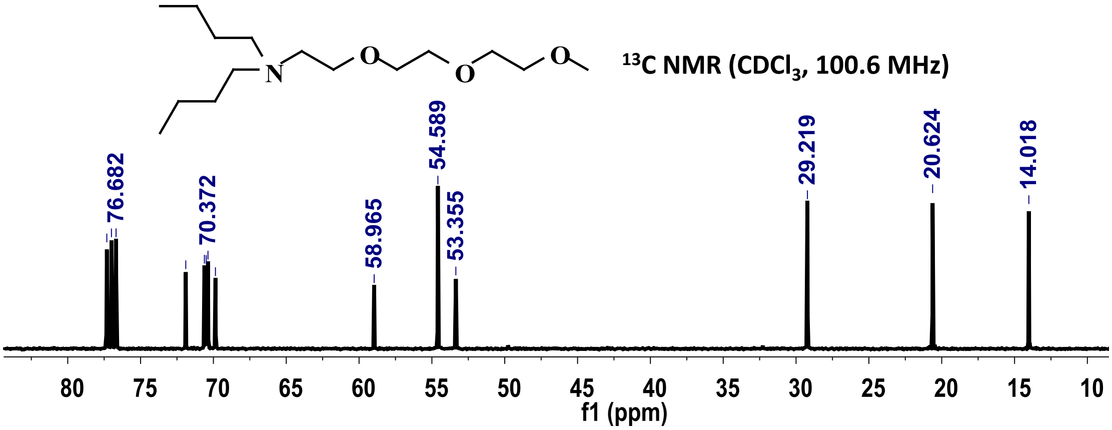

PEG$_{150}$MeBu$_2$N/LiNTf$_2$

^{1}H NMR (CDCl$_3$, 400 MHz) δ 3.70-3.71 (m, 6H), 3.61-3.65 (m, 4H), 3.44 (s, 3H), 2.69 (t, ^3J = 5.2 Hz, 2H), 2.51 (t, ^3J = 8 Hz, 4H), 1.37-1.44 (m, 4H), 1.23-1.32 (m, 4H), 0.92 (t, ^3J = 7.2 Hz, 6H); ^{19}F NMR (CDCl$_3$, 377 MHz) δ -78.9; ^{13}C NMR (CDCl$_3$, 100.6 MHz) δ 124.4, 121.2, 118.0, 114.8, 70.5, 68.5, 68.3, 68.2, 66.8, 59.2, 52.5, 26.7, 20.7, 13.9.

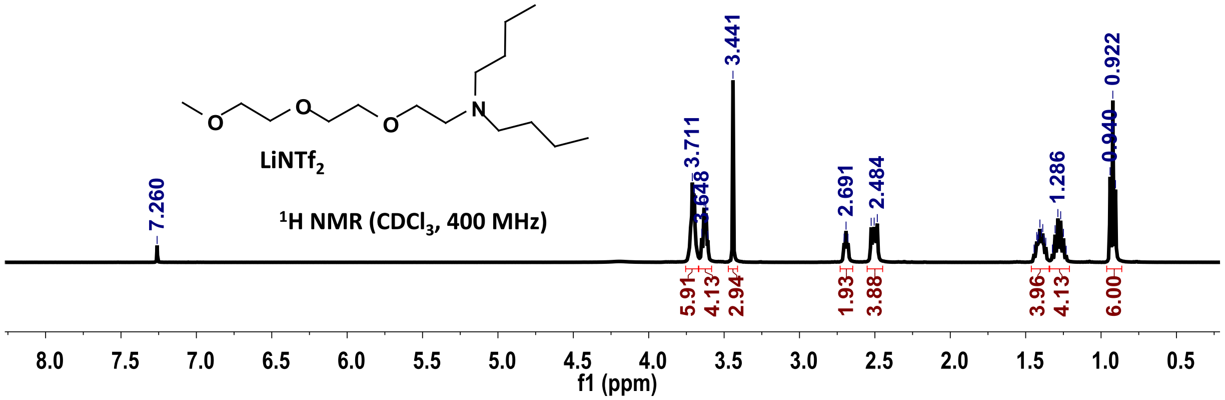

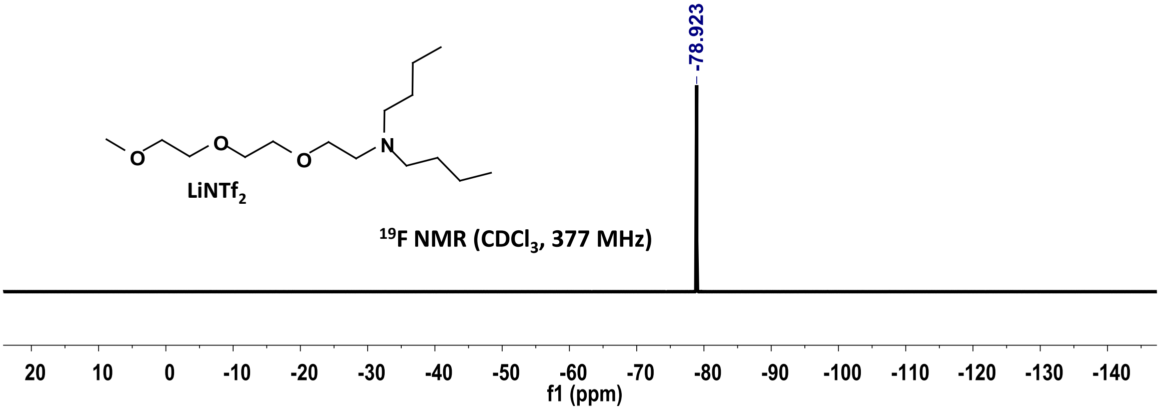

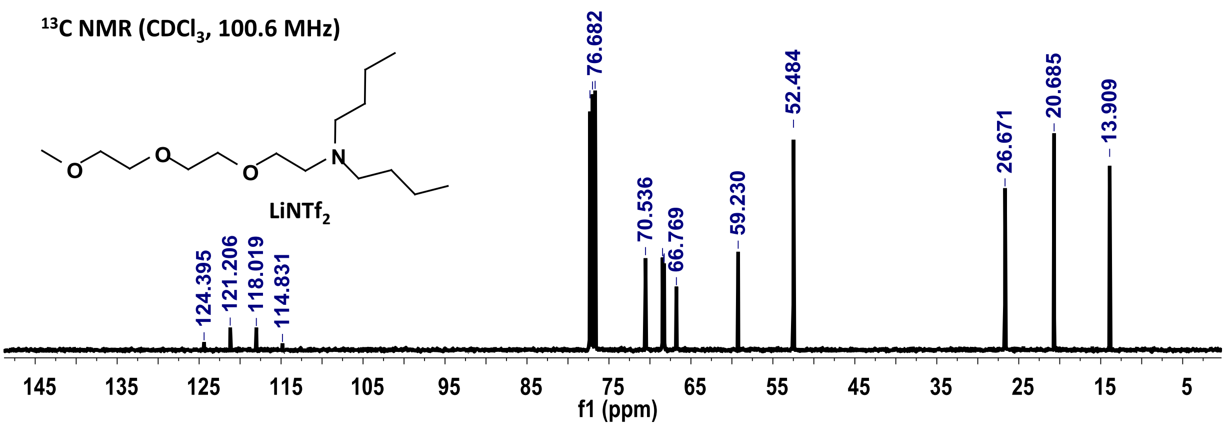

PEG$_{150}$MeBu$_2$N/LiSO$_3$CF$_3$

^{1}H NMR (CDCl$_3$, 400 MHz) δ 3.68-3.73 (m, 6H), 3.59-3.63 (m, 4H), 3.43 (s, 3H), 2.70 (s, 2H), 2.51 (t, ^3J = 7.6 Hz, 4H), 1.37-1.44 (m, 4H), 1.22-1.31 (m, 4H), 0.91 (t, ^3J = 7.2 Hz, 6H); ^{19}F NMR (CDCl$_3$, 377 MHz) δ -78.5; ^{13}C NMR (CDCl$_3$, 100.6 MHz) δ 124.8, 121.6, 118.5, 115.3, 70.7, 69.0, 67.3, 59.2, 52.7, 52.4, 26.8, 20.7, 13.9.

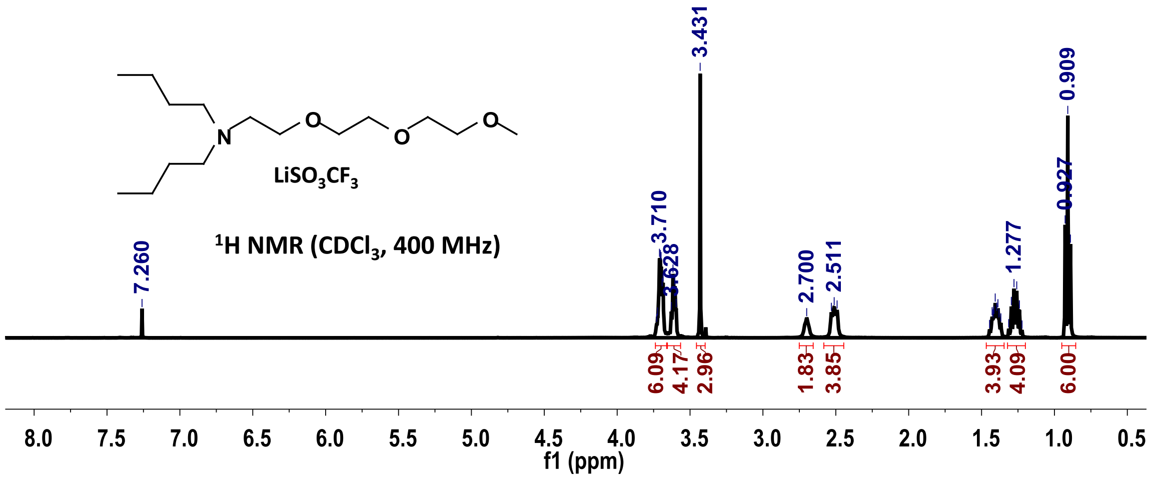

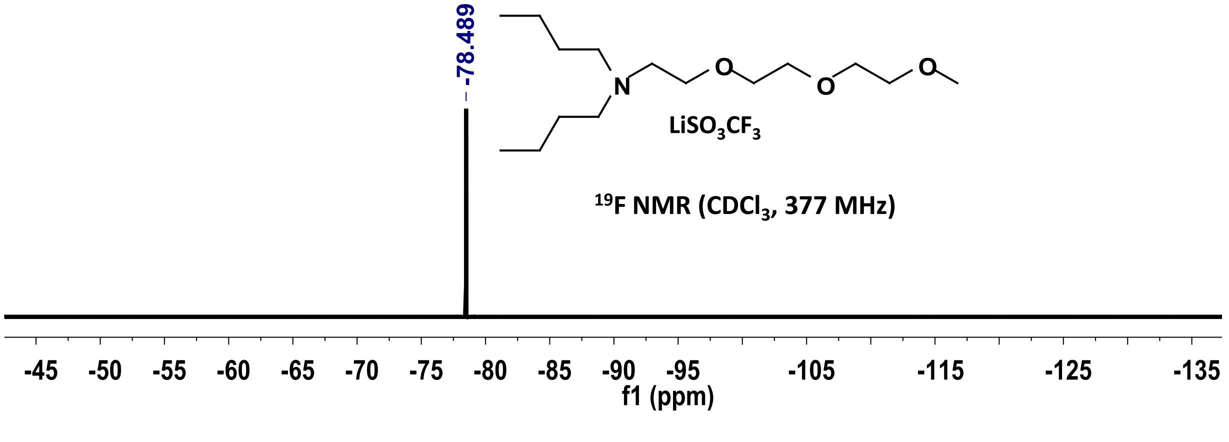

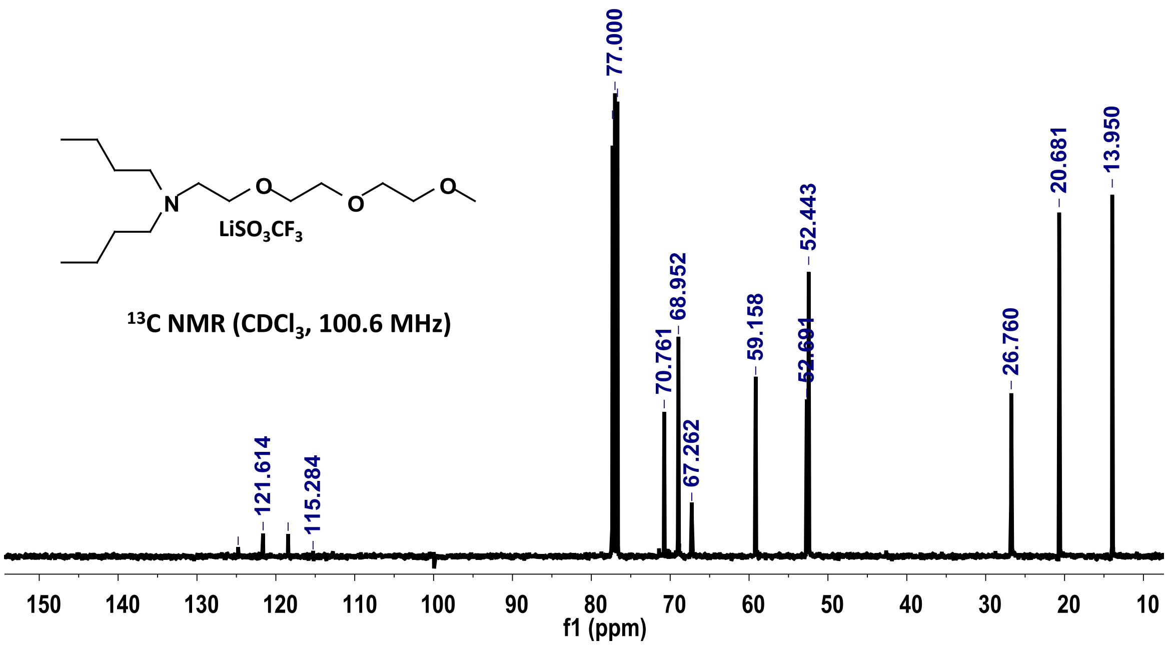

DBU (1,8-diazabicyclo[5.4.0]undec-7-ene):

^{1}H NMR (400 MHz, $CDCl_3$) δ 3.23 (t, ^{3}J = 5.6 Hz, 2 H), 3.13-3.18 (m, 4 H), 2.33-2.35 (m, 2 H), 1.72-1.77 (m, 2 H), 1.60 (s, 4 H), 1.52 (s, 2 H); ^{13}C NMR (100.6 MHz, $CDCl_3$) δ 161.6, 52.8, 48.3, 44.0, 37.2, 29.7, 28.4, 25.9, 22.3.

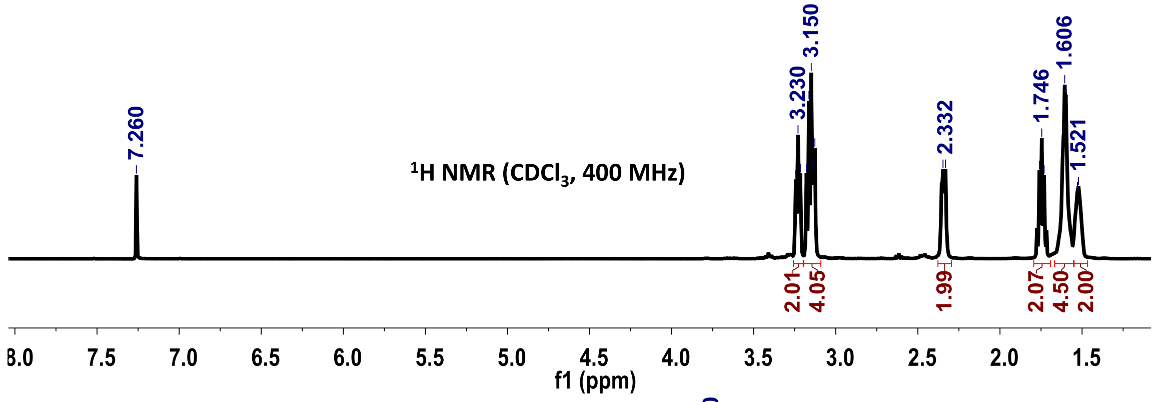

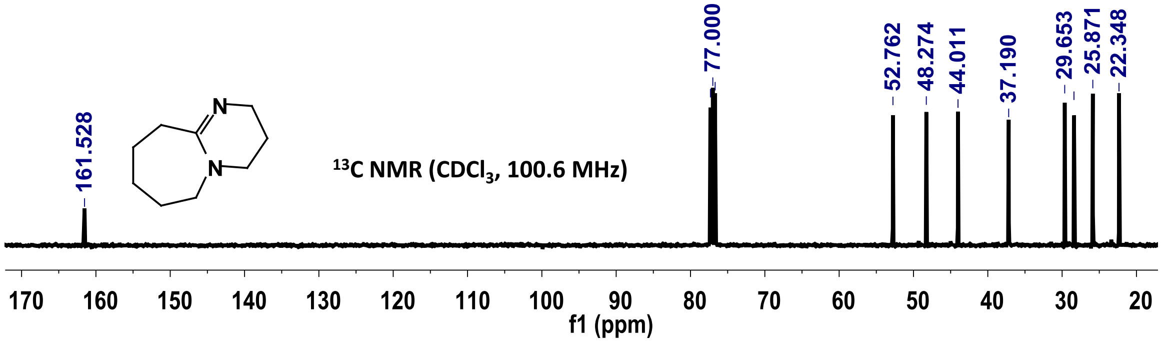

DBU/LiNTf$_2$

^{1}H NMR ($CDCl_3$, 400 MHz) δ 3.28-3.32 (m, 4H), 3.20 (t, 3J = 5.6 Hz, 2H), 2.39-2.42 (m, 2H), 1.81-1.86 (m, 2H), 1.60-1.68 (m, 6H); ^{19}F NMR ($CDCl_3$, 377 MHz) δ -79.2; ^{13}C NMR ($CDCl_3$, 100.6 MHz) δ 164.6, 124.1, 120.9, 117.7, 114.5, 53.5, 48.4, 41.3, 35.3, 29.2, 27.5, 24.8, 21.0.

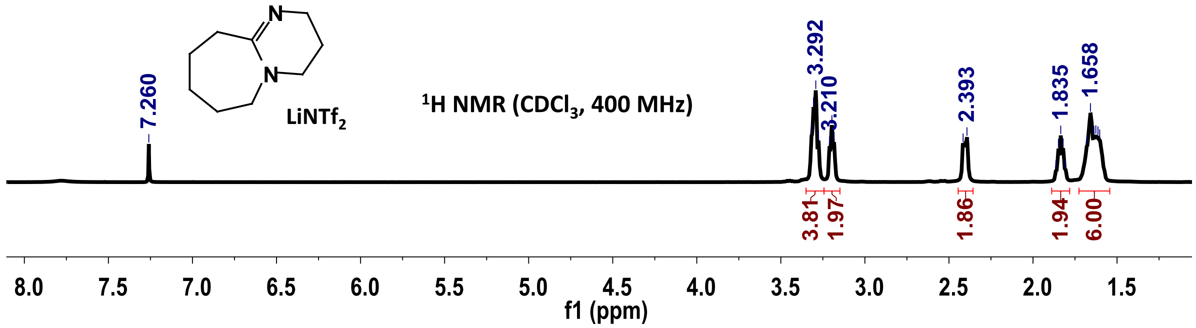

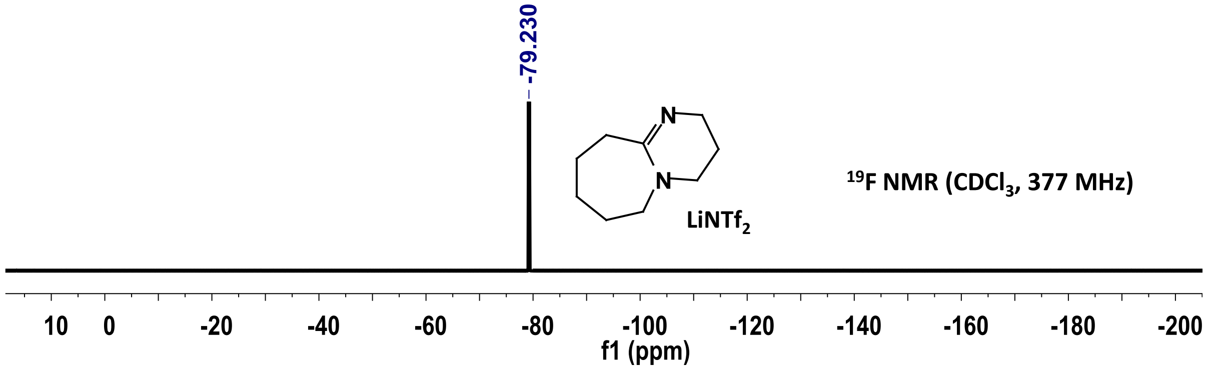

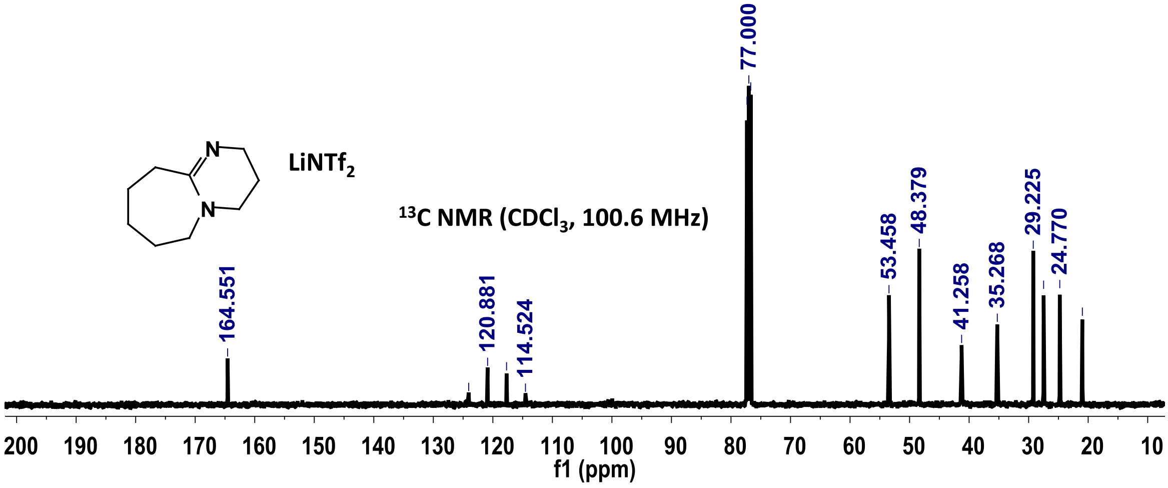

DBN (1,5-diazabicyclo[4.3.0]non-5-ene):

^{1}H NMR (400 MHz, $CDCl_3$) δ 3.28 (t, ^{3}J = 4.8 Hz, 2H), 3.21 (t, ^{3}J = 6.8 Hz, 2H), 3.13 (t, ^{3}J = 6 Hz, 2H), 2.38 (t, ^{3}J = 7.6 Hz, 2H), 1.83-1.91 (m, 2H), 1.70-1.76 (m, 2H); ^{13}C NMR (100.6 MHz, $CDCl_3$) δ 160.5, 51.2, 43.8, 42.8, 31.3, 20.6, 19.4.

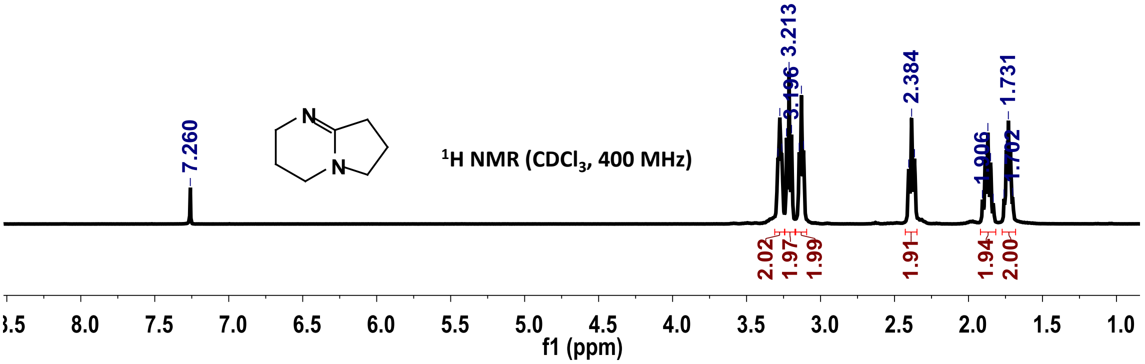

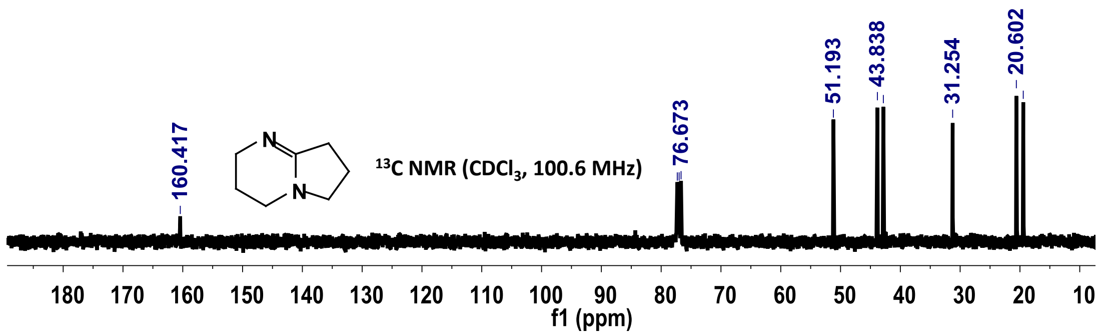

DBN/$LiNTf_2$

^{1}H NMR (400 MHz, $CDCl_3$) δ 3.41 (t, 3J = 7.2 Hz, 2H), 3.25 (t, 3J = 6 Hz, 4H), 2.56 (t, 3J = 8 Hz, 2H), 1.96-2.04 (m, 2H), 1.81-1.87 (m, 2H); ^{19}F NMR ($CDCl_3$, 377 MHz) δ -79.2; ^{13}C NMR (100.6 MHz, $CDCl_3$) δ 163.2, 124.1, 121.0, 117.8, 114.6, 52.3, 42.8, 41.2, 30.6, 19.7, 19.1.

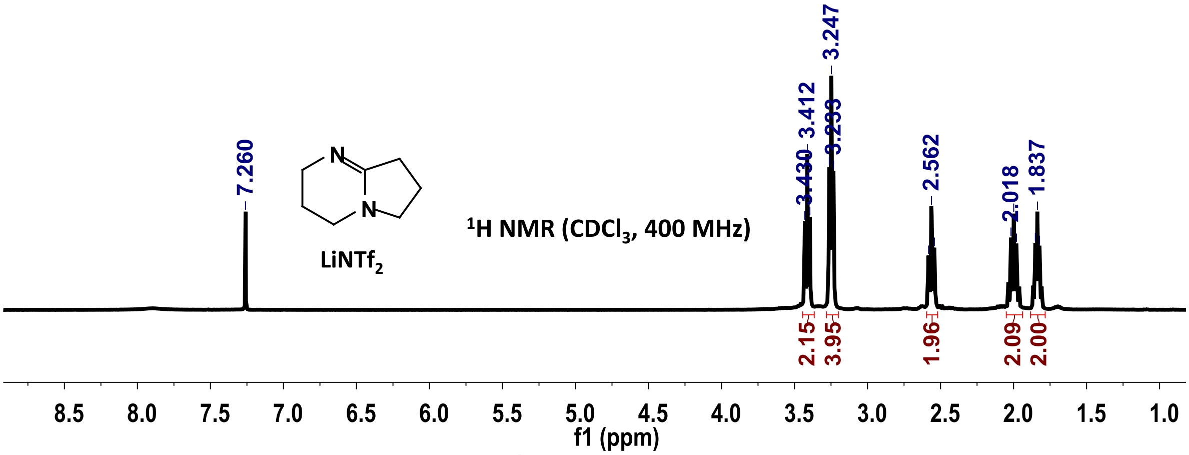

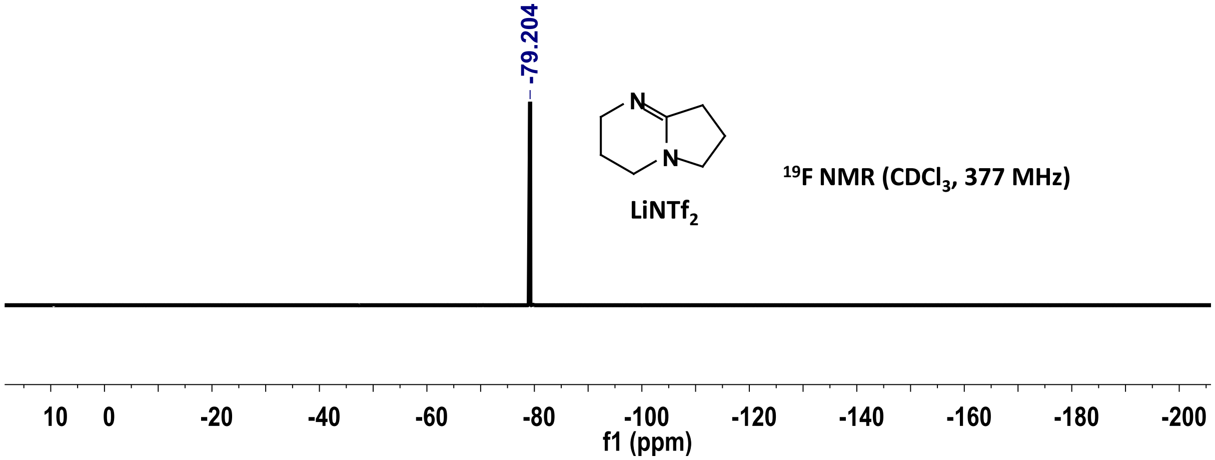

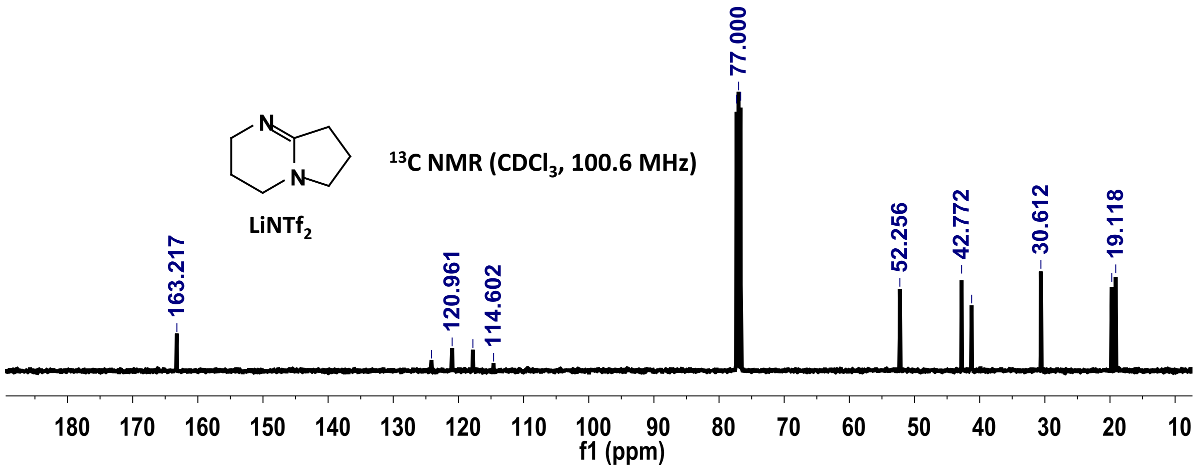

N
N
LiNTf2
13C NMR (CDCl3, 100.6 MHz)
163.217
120.961
114.602
77.000
52.256
42.772
30.612
19.118
180
170
160
150
140
130
120
110
100
90
80
70
60
50
40
30
20
10
f1 (ppm)
